# Triple-junction perovskite–perovskite–silicon solar cells with power conversion efficiency of 24.4%†

**DOI:** 10.1039/d3ee03687a

**Published:** 2024-02-13

**Authors:** Hang Hu, Sophie X. An, Yang Li, Seyedamir Orooji, Roja Singh, Fabian Schackmar, Felix Laufer, Qihao Jin, Thomas Feeney, Alexander Diercks, Fabrizio Gota, Somayeh Moghadamzadeh, Ting Pan, Michael Rienäcker, Robby Peibst, Bahram Abdollahi Nejand, Ulrich W. Paetzold

**Affiliations:** a Institute of Microstructure Technology (IMT), Karlsruhe Institute of Technology (KIT) Hermann-von-Helmholtz-Platz 1 76344 Eggenstein-Leopoldshafen Germany bahram.abdollahi@kit.edu ulrich.paetzold@kit.edu; b Light Technology Institute (LTI), Karlsruhe Institute of Technology (KIT) Engesserstrasse 13 76131 Karlsruhe Germany; c Institute for Solar Energy Research Hamelin (ISFH) Am Ohrberg 1 31860 Emmerthal Germany; d Institute of Electronic Materials and Devices, Leibniz Universität Hannover Schneiderberg 32 30167 Hannover Germany

## Abstract

The recent tremendous progress in monolithic perovskite-based double-junction solar cells is just the start of a new era of ultra-high-efficiency multi-junction photovoltaics. We report on triple-junction perovskite–perovskite–silicon solar cells with a record power conversion efficiency of 24.4%. Optimizing the light management of each perovskite sub-cell (∼1.84 and ∼1.52 eV for top and middle cells, respectively), we maximize the current generation up to 11.6 mA cm^−2^. Key to this achievement was our development of a high-performance middle perovskite sub-cell, employing a stable pure-α-phase high-quality formamidinium lead iodide perovskite thin film (free of wrinkles, cracks, and pinholes). This enables a high open-circuit voltage of 2.84 V in a triple junction. Non-encapsulated triple-junction devices retain up to 96.6% of their initial efficiency if stored in the dark at 85 °C for 1081 h.

## Abstract 2

Key advances on triple-junction perovskite–perovskite–Si solar cells with an unprecedented efficiency of 24.4% and enhanced long-term thermal stability are reported *via* the development of high-performance middle perovskite solar cell.

## Graphical abstract

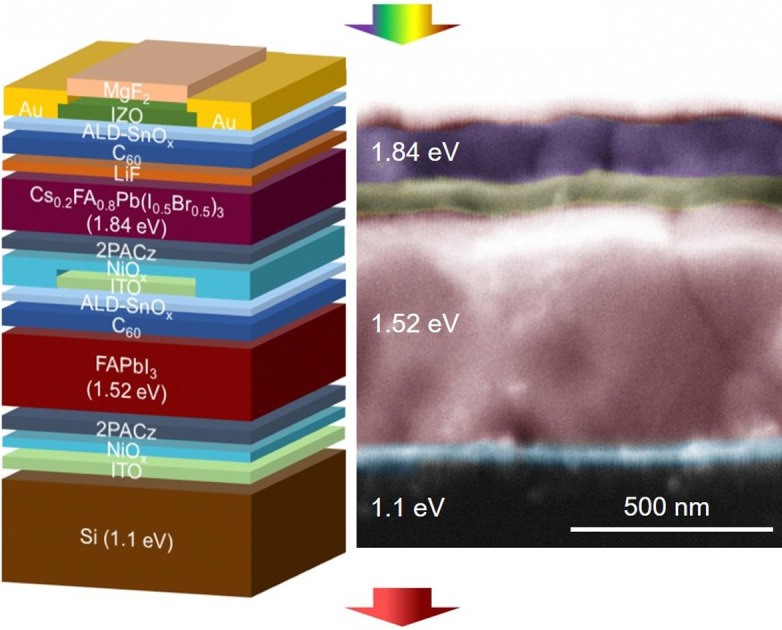

**Broader context**

Metal halide perovskite semiconductors are the prime candidate for next generation of ultra-high-efficiency multi-junction photovoltaics (PVs) using three or even more junctions. However, triple-junction PVs (*e.g.*, perovskite–perovskite–silicon) lag far behind in performance with only very few reports on prototypes. One of the key challenges in processing triple junction to date is the most critical junction, *i.e.*, middle perovskite sub-cell, since it is processed on top of the silicon bottom cell and needs to withstand the subsequent processing of the wide-bandgap perovskite top cell. This work presents key advances on triple-junction perovskite–perovskite–silicon solar cells with a record efficiency of 24.4%. Key achievements are developing a stable pure-α-phase high-quality middle perovskite thin film and optimizing the light management of each perovskite sub-cell. This work opens the door to a new era of perovskite-based high-efficiency triple-junction PVs.

## Introduction

Recent advancements in power conversion efficiencies (PCEs) of monolithic perovskite-based double-junction solar cells^1–8^ denote just the start of a new era in ultra-high-efficiency multi-junction photovoltaics (PVs) using three or even more junctions. Such devices will surpass by far the detailed-balanced limit in PCE for single-junction devices^9^ and might even compete at one stage with triple- and six-junction solar cells based on epitaxially grown III–V crystalline semiconductor thin films,^1,10^ given the bandgap tunability, excellent optoelectronic characteristics, and low-cost facile process of perovskite material class.^11,12^ Incorporation of a third sub-cell into a double-junction stack, *i.e.*, triple-junction architecture, can further increase energy yield (*e.g.*, reducing thermalization loss).^9,11,13^ The detailed balance limit for triple-junction PVs of 51% (compared to ∼45% for double junctions) sets the ultimate boundary and shows the headroom for next-generation perovskite-based multi-junction PVs.^14,15^ However, as high-performance perovskite semiconductors are only available today for bandgaps down to ∼1.25 eV,^1,6–8^ monolithic perovskite-based multi-junctions will need to be combined with a narrow bandgap silicon (Si) or a copper indium (gallium) selenide bottom solar cell – *e.g.*, in a perovskite–perovskite–Si architecture – to exploit the solar spectrum efficiently.

To reach these high performances, several challenges need to be addressed, such as the sequential processing of high-quality perovskite thin films in the increasingly complex multi-layer architecture, light management, and current matching of the monolithically interconnected sub-cells, as well as the development of low-loss tunnel/recombination junctions. Optical modeling reveals potential in PCEs of up to 36.6% and 38.8% for all-perovskite and perovskite–perovskite–Si monolithic triple-junction solar cells (MTJSCs), respectively.^11^ However, the experimental realization lags far behind and, to date, only very few prototypes of all-perovskite^14,16–19^ or perovskite-perovskite–Si MTJSCs^12,15,20,21^ were demonstrated, reaching maximum PCEs of 25.1%^19^ and 22.2%,^20^ respectively. The device performances of perovskite–perovskite–Si MTJSCs were limited with imperfect recombination junction between perovskite sub-cells (*i.e.*, either low fill factor (FF) or low open-circuit voltage (*V*_OC_))^12^ or low current generation in the middle perovskite sub-cell (*i.e.*, low short-circuit current density (*J*_SC_) <10.2 mA cm^−2^).^12,15,20,21^ It is highlighted that to date the most critical junction in the sequential processing of perovskite–perovskite–Si MTJSC is the middle-bandgap (MBG) perovskite sub-cell, since it is processed on top of the Si bottom cell and needs to withstand the subsequent processing of the wide-bandgap (WBG) perovskite top cell. The middle perovskite sub-cell is required to provide (i) a suitable MBG, (ii) very good thermal stability, (iii) excellent interfaces to both recombination junctions, and (iv) a low density of defects and pinholes. Optical modeling and simulations predict that MBG perovskite with a bandgap of 1.40–1.50 eV is favored to maximize *J*_SC_ in a triple-junction perovskite–perovskite–Si architecture.^11,12^ We note that – to date – high-quality perovskite thin films with bandgaps <1.50 eV rely on unstable mixed Sn/Pb-based compositions that degrade swiftly if exposed to minimal amounts of oxygen and water.^6,7^ For this reason, only Pb-based compositions of Cs_*x*_FA_*y*_MA_1−*x*−*y*_Pb(I_*z*_Br_1−*z*_)_3_ (Cs: cesium; FA: formamidinium; MA: methylammonium; Pb: lead; I: iodide; Br: bromide) were used in perovskite–perovskite–Si MTJSCs.^12,15,20,21^ The most promising candidate for a high-efficiency and reasonably stable MBG perovskite thin film is FAPbI_3_, which exhibits an optical bandgap of ∼1.52 eV^22–24^ (desired for (i)). Single-junction FAPbI_3_ perovskite solar cells have demonstrated excellent device performance and offer good thermal stability^22–28^ (addressing (ii)) but have not yet been employed in multi-junction PVs. Moreover, (iii) and (iv) are crucial to prevent degradation of the multi-layer thin-film stacks during subsequent solution-based processing of the WBG perovskite top solar cell (see our previous reports of all-perovskite double-junction modules^7^). Since conventional anti-solvent (AS) quenching methods are reported to induce surface wrinkles, micro-cracks, and/or pinholes,^29–32^ as well as high density of defects at the perovskite/electron transport layer (ETL) interface,^32,33^ an alternative AS-free quenching method for processing the MBG perovskite thin film is highly encouraged.

In this work, we present high-efficiency perovskite–perovskite–Si MTJSCs using a Cs_0.2_FA_0.8_Pb(I_0.5_Br_0.5_)_3_ WBG perovskite top cell (∼1.84 eV), a FAPbI_3_ perovskite middle cell (∼1.52 eV), and a Si bottom cell (∼1.1 eV). Our champion device achieves an unprecedented PCE of 24.4%, which is, to our knowledge, the highest PCE (active area of ∼0.5 cm^2^) reported for this architecture. *Via* optimizing light management and maximizing the photocurrent of the perovskite sub-cells, we further improve the *J*_SC_ up to 11.6 mA cm^−2^, which exceeds previous reports.^12,15,20,21^ Importantly, using a vacuum-assisted growth (VAG) control, we process high-quality (free of wrinkles, pinholes, and cracks) and phase-stable FAPbI_3_ middle perovskite sub-cell. Our perovskite–perovskite–Si solar cells demonstrate high *V*_OC_ up to 2.84 V, given high-quality thin films and low non-radiative recombination loss at the perovskite/ETL interfaces. Moreover, non-encapsulated triple-junction solar cells show good thermal stability and retain 96.6% of initial PCE if stored in an N_2_ atmosphere at 85 C for 1081 h.

## Results and discussion

### Simulations of perovskite–perovskite–Si MTJSCs

**Fig. 1:**
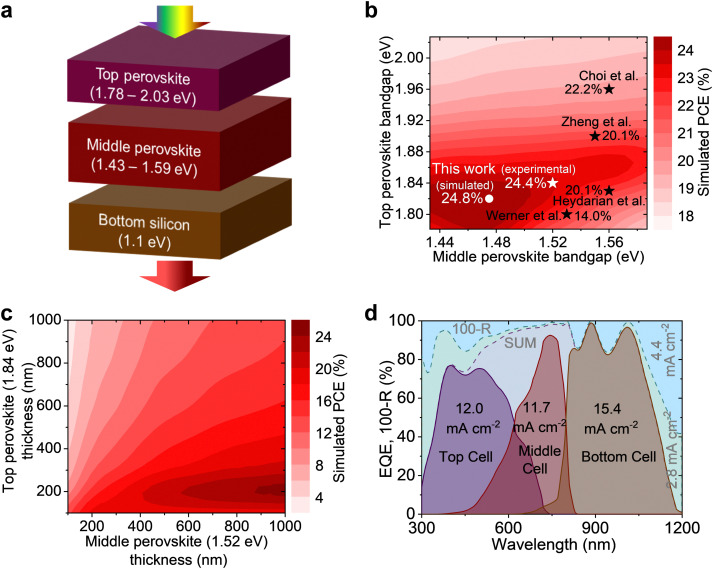
Simulations of perovskite–perovskite–Si MTJSCs. (a) Illustration of triple-junction concept. Contour plots of simulated PCEs of perovskite–perovskite–Si MTJSCs for (b) varied bandgap combinations of top/middle perovskites and (c) varied thickness of top (1.84 eV) and middle (1.52 eV) perovskites. In (b), the black stars indicate experimental PCEs from the reports,^12,15,20,21^ and the white dot (star) indicates simulated (experimental) PCEs from this work. (d) Simulated EQE spectra for the MTJSC combined with perovskite bandgaps of 1.84 eV and 1.52 eV. The parasitic absorption and reflection losses are calculated to be 2.8 mA cm^−2^ and 4.4 mA cm^−2^, respectively.

To maximize the overall *J*_SC_ in our perovskite–perovskite–Si triple-junction architecture, we perform numerical simulations with the target to minimize parasitic absorption and reflection losses and to identify the optimal combination of bandgaps as well as thicknesses of the perovskite thin films for the top and middle sub-cells (the bandgap of the Si bottom cell is fixed ∼1.1 eV, Fig. 1(a)). The device architecture is based on prototypes reported in literature with realistic experimental data (see experimental procedures for details) and established material combinations used in our previous work for double-junction architectures.^7,34,35^ Our simulations predict – for the perovskite–perovskite–Si triple-junction architecture under study (Fig. 1(a)) – a maximum PCE of 24.8% for the ideal bandgap combination of ∼1.82 eV and ∼1.48 eV of the top and middle perovskite sub-cells, respectively (Fig. 1(b) and Fig. S1, S2, ESI†). Previous reports on perovskite–perovskite–Si MTJSCs applied bandgap combinations that differ significantly for this optimum. For instance, Zheng *et al.* reported in 2022 a PCE of 20.1% with a *J*_SC_ of 8.5 mA cm^−2^ using a bandgap combination of 1.55 eV and 1.90 eV,^15^ and Choi *et al.* latest reported a PCE of 22.2% with a *J*_SC_ of 10.19 mA cm^−2^ using a bandgap combination of 1.56 eV and 1.96 eV.^20^ The current generation in these devices was constrained by the middle sub-cell. As discussed above, except for Sn/Pb mixed perovskite compositions, we compromise in this study to a small extent on the maximum achievable efficiency, thereby extending the range of suitable bandgap combinations. FAPbI_3_ provides a suitable optical bandgap ∼1.52 eV^25–28^ (∼1.48 eV is also reported as an ideal bandgap of pure black cubic α-phase FAPbI_3_^22–24^ which is very close to our optimal simulated value), excellent performance, and thermal stability in single-junction devices.^22–28^ We note that good charge carrier extraction was achieved in our previous experimental studies for perovskite solar cells based on up to ∼600 nm thick planar FAPbI_3_ absorber.^36^ According to our simulations, the ideal counterpart for a >600 nm thick FAPbI_3_ middle sub-cell is a perovskite top sub-cell with a WBG of ∼1.84 eV (Fig. 1(c) and Fig. S3–S5, ESI†) and an absorber thickness of ∼200 nm. The thinner (<200 nm) or thicker (>300 nm) WBG perovskite layer of the top cell would result in a significant current mismatch between these two perovskite sub-cells, *i.e.*, as a consequence of an overall *J*_SC_ < 10 mA cm^−2^ and significant loss in PCE (see Fig. S4 and S5, ESI†). Fig. 1(d) shows a close to perfect current matching between the top and middle cub-cells in the champion simulated perovskite (∼1.84 eV)–perovskite (∼1.52 eV)–Si (∼1.1 eV) MTJSCs. The simulated integrated photocurrent densities are 12.0, 11.7, and 15.4 mA cm^−2^ for the top, middle, and bottom sub-cells, respectively (Fig. 1(d)). The parasitic absorption and reflection losses could be minimized to 7.2 mA cm^−2^ in total. The latter leaves room for improvement in future works.

### PV performance of perovskite–perovskite–Si MTJSCs

**Fig. 2:**
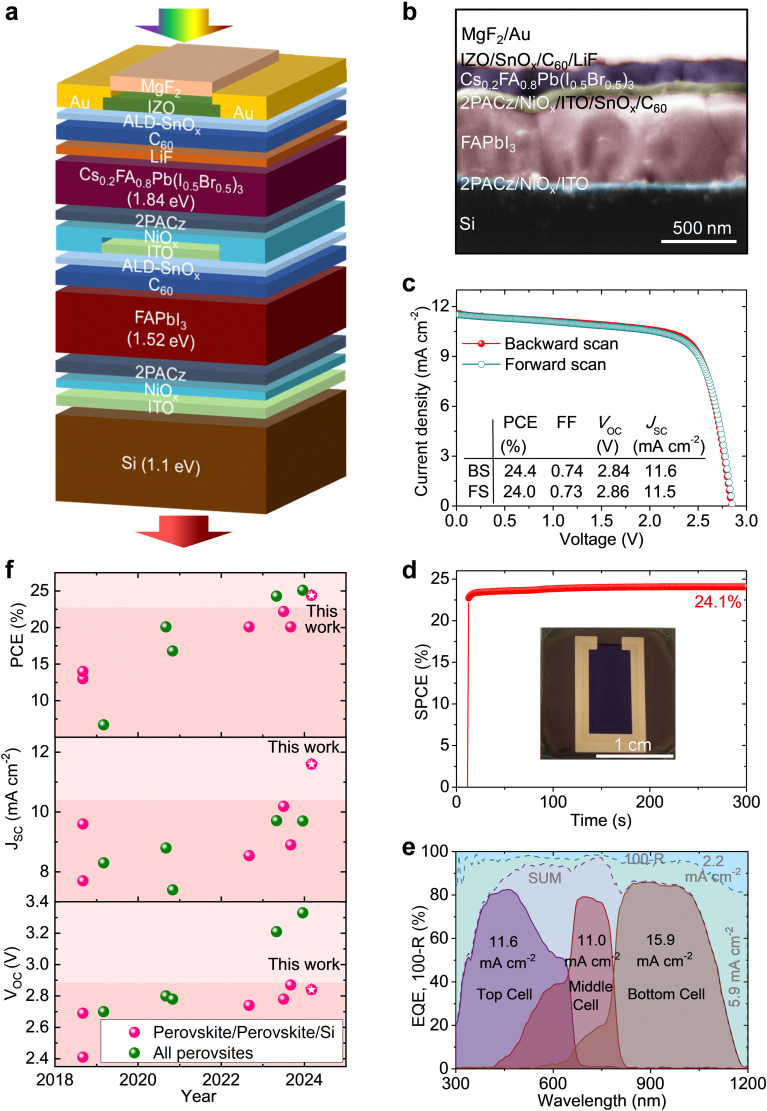
PV performance of perovskite–perovskite–Si MTJSCs. (a) Schematic and (b) cross-section SEM image of perovskite–perovskite–Si triple-junction architecture. (c) *J*–*V* characteristics of forward and backward scans, (d) stabilized PCE under continuous AM 1.5G illumination, and (e) EQE spectra of a champion perovskite–perovskite–Si MTJSC. The parasitic absorption and reflection losses are calculated to be 5.9 mA cm^−2^ and 2.2 mA cm^−2^, respectively. (f) Summary of reported perovskite-based MTJSCs.^12,14–21^

Perovskite–perovskite–Si MTJSCs were fabricated according to the above-described rationale (see Fig. 2(a)). The layer stack of the top and middle junction perovskite solar cells was processed on top of a ∼200-μm-thick potassium hydroxide-etched Si bottom solar cell (with passivating electron-selective full-area front and hole-selective-partial textured rear contacts based on a doped polysilicon-on-oxide (POLO) contact scheme). The entire layer stack of middle and top perovskite sub-cells is indium tin oxide (ITO)/nickel oxide (NiO_*x*_)/[2-(9*H*-carbazol-9-yl)ethyl]phosphonic acid (2PACz)/FAPbI_3_/fullerene (C_60_)/tin oxide (SnO_*x*_)/ITO/NiO_*x*_/2PACz/Cs_0.2_FA_0.8_Pb(I_0.5_Br_0.5_)_3_/lithium fluoride (LiF)/C_60_/SnO_*x*_/indium zinc oxide (IZO)/gold (Au)/magnesium fluoride (MgF_2_). Fig. 2(b) shows the triple-junction solar cell's cross-sectional scanning electron microscopy (SEM) image. For the middle and top perovskite sub-cells, perovskite thin films with the nominal compositions of FAPbI_3_ (∼1.52 eV, see Fig. S6, ESI;† ∼650 nm) and Cs_0.2_FA_0.8_Pb(I_0.5_Br_0.5_)_3_ (∼1.84 eV, see Fig. S7, ESI;† ∼200 nm) were applied, respectively. The recombination junctions are formed by sputtered ITO layers (15–20 nm). We note that ITO also serves as anchoring oxide for the sequential hole transport layer (HTL), especially for the double HTLs of NiO_*x*_/self-assembled monolayer (SAM).^3^ A double HTLs based on a combination of sputtered NiO_*x*_ and 2PACz is used in both perovskite subcells, offering an excellent charge carrier extraction, a robust barrier for the solvents of perovskite precursor, and a very good yield for the devices.^12,15,17^ Our champion device provides a PCE of 24.4% (backward scan, *V*_OC_: 2.84 V, *J*_SC_: 11.6 mA cm^−2^, FF: 0.74) with negligible hysteresis in current-density–voltage (*J*–*V*) characteristics (see Fig. 2(c)). After 5 min of continuous maximum power point (MPP) tracking under continuous AM 1.5G (100 mW cm^−2^) irradiation the solar cell shows 24.1% (Fig. 2(d)) of PCE. To our knowledge, this is the highest reported PCE for perovskite–perovskite–Si MTJSCs. We highlight that the perovskite layer of the middle sub-cell is processed with the VAG strategy in this work, enabling higher PV performance and yield in triple junctions compared to using a conventional AS method (see Fig. S8 and S9, ESI,† backward scan, PCE: 15.9%, *V*_OC_: 2.65 V, *J*_SC_: 10.9 mA cm^−2^, FF: 0.55). Our champion perovskite–perovskite–Si MTJSC exhibits a similar external quantum efficiency (EQE) to the simulated optimum (Fig. 1(d)) with integrated photocurrent densities of 11.6, 11.0, and 15.9 mA cm^−2^ for the top perovskite sub-cell (∼1.84 eV), the middle perovskite sub-cell (∼1.52 eV), and the Si bottom sub solar cell (∼1.1 eV) (see Fig. 2(e)), respectively. Given the low-loss transparent and conductive oxide front electrode (TCO, Fig. S10a, ESI†) and the optimized antireflection coating (using MgF_2_, Fig. S10b and c, ESI†) layers the device shows low absorption losses (corresponding photocurrent density ∼5.9 mA cm^−2^, Fig. 2(e)) and reflection losses (corresponding photocurrent density ∼2.2 mA cm^−2^, Fig. 2(e)). We highlight that the remarkable device performance of our MTJSCs stands out from the very few previous reports for this type of solar cells (Fig. 2(f)). Our device exceeds previous prototypes of perovskite–perovskite–Si MTJSCs in *J*_SC_ (Fig. 2(f)).

The major challenge in processing the entire stack of our MTJSCs arises from the large number of subsequent layer depositions. While most of the layers in our architecture are processed by physical vapor deposition, which allows for controlled conformal deposition of thin films, the perovskite absorber layers are spin-coated. To avoid a penetration of the solvents (dimethylformamide (DMF)/dimethyl sulfoxide (DMSO)) and anti-solvents (ethyl acetate (EA)) in the underlying layers of the device architecture and to allow for the processing of pinhole-free perovskite thin films, we employ the VAG strategy established in our team for the processing of all-perovskite double-junction solar cells^6,7^ and high-quality FAPbI_3_ thin films.^36^ VAG process achieves a significantly higher performance (Fig. S11, ESI†) in a semitransparent single-junction middle cell (PCE: 20.0%, *V*_OC_: 1.08 V, FF: 0.84, *J*_SC_: 22.0 mA cm^−2^) compared to AS method (PCE: 13.9%, *V*_OC_: 1.00 V, FF: 0.66, *J*_SC_: 21.1 mA cm^−2^). The thickness of MBG FAPbI_3_ thin films is optimized (up to ∼650 nm) by controlling the concentration of perovskite precursor (*i.e.*, optimum 1.3 M, see Fig. 2(b)). A thinner MBG perovskite layer leads to insufficient light absorbing, resulting in a lower *J*_SC_ (see Fig. S12, ESI†) in single junction as well as a current mismatch in triple junctions (Fig. S13, ESI†). A thicker MBG perovskite layer impacts the charge extraction. It leads to the increased non-radiative recombination,^37^ which impacts *V*_OC_ and FF (see Fig. S12, ESI†) and leads to a current mismatch in triple junction (Fig. S13a and b, ESI†). Compared to the AS method, the increase in *J*_SC_ in the VAG-processed middle cell is pivotal to realizing a good current match between the top and middle sub-cells in the triple-junction architecture (Fig. 2(e) and Fig. S13, S10, S11b, and c, ESI†). We attribute the *V*_OC_ enhancement in the middle sub-cell processed by VAG to the reduced non-radiative recombination loss, which is discussed later.

For the top cell, the thickness of WBG perovskite is optimized to ∼200 nm (Fig. 2(b) and Fig. S4, S5, and S13, ESI†) in triple junctions. We find that AS and VAG methods deliver similar PV performance in the semitransparent architecture of the top cell (Fig. S14 and S15, ESI†). We hypothesize that (i) the faster nucleation/crystallization of the Br-rich WBG perovskites thin film^38^ and/or (ii) the promotion of the photoactive (black) phase formation due to the presence of Br^−^ during the nucleation^39^ facilitates the formation of WBG perovskite thin film compared to MBG perovskite thin film. Future studies on the perovskite thin film formation during the AS and VAG methods for processing high-performance WBG perovskite PVs are required to shed light on this subject, and we believe that novel *in situ* characterization methods will be valuable tools to study these aspects.

### Morphology and phase stability of middle perovskite thin films

**Fig. 3:**
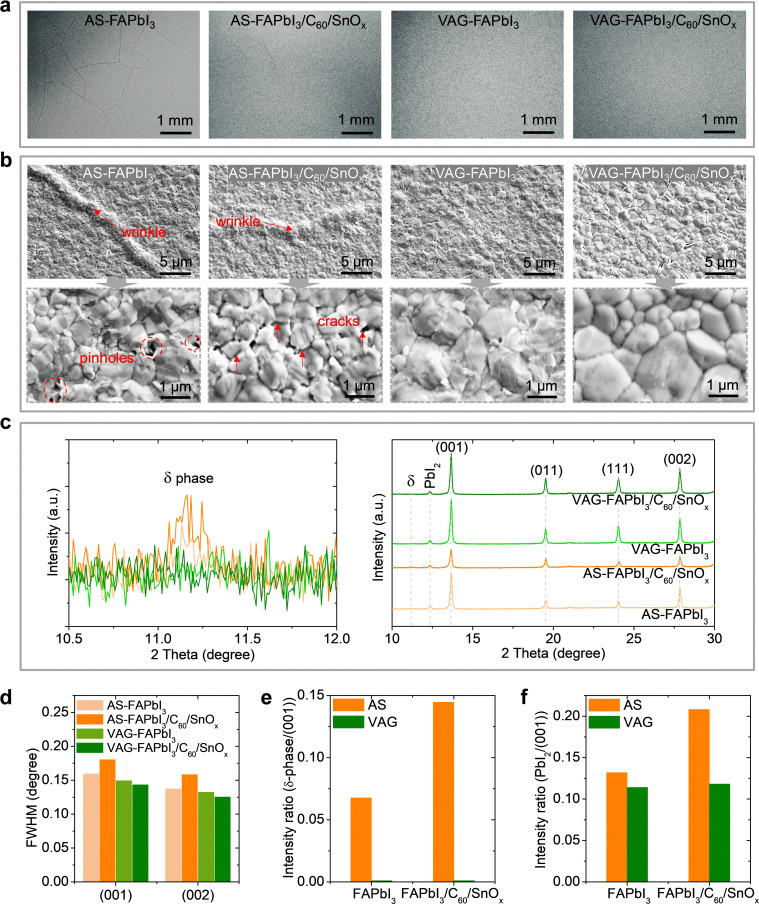
Morphology and phase stability of middle perovskite thin films. (a) Top-view optical microscopic and (b) SEM images of middle perovskite thin films and sequential deposition of C_60_ and SnO_*x*_ layers. FAPbI_3_ perovskite thin films were fabricated with VAG and AS methods. (c) XRD patterns and (d) the corresponding full width at half maximum (FWHM). The XRD intensity ratios of (e) δ-phase/(001) plane and (f) PbI_2_/(001) plane for FAPbI_3_ thin films and FAPbI_3_/C_60_/SnO_*x*_ stack.

The quality of the perovskite thin films, *i.e.*, the middle-junction FAPbI_3_, is decisive for the performance.^29,32^ In this regard, we examine the morphology of FAPbI_3_ thin films fabricated by AS and VAG methods (referred to as AS-FAPbI_3_ and VAG-FAPbI_3_) and the sequential layers with C_60_ and SnO_*x*_ (*i.e.*, the layer stack of AS-FAPbI_3_/C_60_/SnO_*x*_ and VAG-FAPbI_3_/C_60_/SnO_*x*_). We observe micro-wrinkles throughout the surface of AS-FAPbI_3_ and AS-FAPbI_3_/C_60_/SnO_*x*_ thin films, as shown in the microscopic and SEM images (Fig. 3(a) and (b)). Such surface wrinkles have been reported in the processing of a wide range of perovskite thin films.^29–32^ They are caused by the relaxation of the compressive strain during perovskite crystallization.^29–31,33^ Since the thermal expansion coefficient for TCO or glass substrate is one order of magnitude lower than perovskite and organic thin films,^40^ the substrate (*i.e.*, Si substrate/ITO/NiO_*x*_/2PACz layer stack in this work) constraints the perovskite volume expansion during the intermediate phase formation, resulting in energy release of compressive strain. In the AS quenching process, the anti-solvent (*i.e.*, EA) assists in removing solvents (*i.e.*, DMF/DMSO), but the extraction rate is not sufficient during the formation of an intermediate-phase thin film, resulting in compressive strain release.^30^ In contrast, during the VAG process, the prompt extraction and well-controlled removal of precursor solvents (*i.e.*, DMF/DMSO) lead to a faster formation intermediate phase^41,42^ (*i.e.*, the intermediate phase is formed after ∼20 s during the VAG process, optimal vacuum time: 30 s). After post-annealing, less solvent releases or less perovskite volume changes between the perovskite thin film and the underlying substrate, resulting in less or free residual strain.^33^ The remaining cracks (Fig. 3(b) and Fig. S16, ESI†) in AS-FAPbI_3_/C_60_/SnO_*x*_ stack indicate significant residual strain releasing during the temperature-dependent atomic layer deposition of SnO_*x*_ thin film (ALD-SnO_*x*_, *i.e.*, 90 °C deposition for 2.5 h for ∼30 nm^5,7^ in this work). Notably, the pinholes or cracks observed in AS-FAPbI_3_ thin film (Fig. S17, ESI†) allow for faster penetration of the solvents of the WBG perovskite^7^ (Fig. S18, ESI†), resulting in non-radiative recombination centers^33^ or shunting in a single-junction as well as a triple-junction architecture (see Fig. S8, ESI†).

One of the aims of using the VAG process is to obtain stable α-phase FAPbI_3_ thin films. X-ray diffraction (XRD) patterns confirm α-phase formed in FAPbI_3_ thin films^43^ for both routes of fabrication (Fig. 3(c)). Compared to AS-FAPbI_3_ thin films, the reduction of full width at half maximum (FWHM) observed in all VAG-FAPbI_3_ thin films (even after deposition of C_60_/SnO_*x*_ layers) reflects a larger size of crystallites (Fig. 3(d) and Fig. S19, ESI†). Most importantly, the yellow orthorhombic phase (δ-FAPbI_3_) emerges in AS-FAPbI3 thin film (Fig. 3(c)), and α-to-δ phase transition occurs (Fig. 3(e)) after sequential deposition of C_60_/SnO_*x*_, as the ratio of δ/(001) increases further (almost double). The residual strain may induce a pronounced α-to-δ phase transition as the cracks and wrinkles are still found in the AS-FAPbI_3_/C_60_/SnO_*x*_ stack (see Fig. 3(a) and (b)).^26,44,45^ The strain releasing in AS-FAPbI_3_ thin film during the ALD process causes cracks in the C_60_ layer as well, generating a penetration pathway for diffusion of the tetrakis(dimethylamino)tin(iv) precursor during ALD-SnO_*x*_ deposition and consequently reaction with AS-FAPbI_3_ layer. In contrast, FAPbI_3_ thin films fabricated by VAG process remain pure α phase before and after sequential deposition, demonstrating superior phase stability. Furthermore, an apparent PbI_2_ peak is found in all FAPbI_3_ thin films (Fig. 3(f)) as 10 mol% of excess PbI_2_ is utilized as a standard recipe in this work, which has been reported in many FA-based perovskite solar cells with a p–i–n architecture.^36,46,47^ The peak ratio of PbI_2_/(001) retains ∼11% for both of VAG-FAPbI_3_ thin film and VAG-FAPbI_3_/C_60_/SnO_*x*_ stack, whereas AS-FAPbI_3_/C_60_/SnO_*x*_ stack delivers an increased ratio from ∼13% to >20%. This reveals no degradation in the VAG process and during sequential layer depositions (especially in the ALD process) but accelerated degradation to PbI_2_ for AS-FAPbI_3_ thin film during the ALD process that may also be caused by the residual strain.^44^

In brief, the VAG process enables high-quality and stable pure-α-phase MBG FAPbI_3_ thin films. This provides an ideal substrate for two sequential rounds of the ALD-SnO_*x*_ process to protect the MBG perovskite against the high-energy sputtering for recombination junction (Fig. 3(a), (b) and Fig. S16, S17, ESI†), against solvent corrosion (*i.e.*, degradation or dissolution, Fig. S18, ESI†) for WBG (1.84 eV) processing, and high-temperature post annealing (*e.g.*, ∼150 °C for WBG). This enhancement contributes to high PV performance in a triple-junction architecture (Fig. 2).

### Characteristics of non-radiative recombination for middle sub-cell

To elucidate the origin of the *V*_OC_ improvement (*e.g.*, in the perovskite bulk or/and at the interfaces), we investigate the middle perovskite thin films and single-junction devices with following three architectures: (i) a half stack of ITO/2PACz/FAPbI_3_, (ii) a whole stack of ITO/2PACz/FAPbI_3_/C_60_/SnO_*x*_, and (iii) full opaque or semitransparent device.

**Fig. 4:**
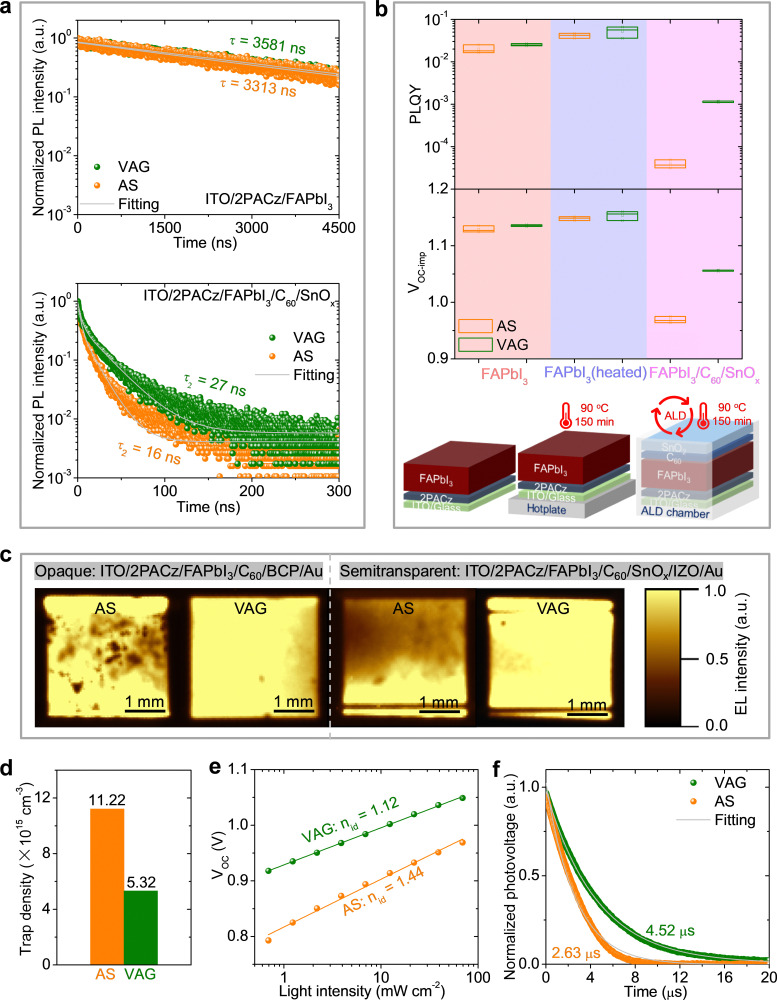
Characteristics of non-radiative recombination for middle sub-cell. (a) TRPLs and (b) PLQYs for ITO/2PACz/FAPbI_3_ and ITO/2PACz/FAPbI_3_/C_60_/SnO_*x*_ stacks. (c) EL imaging of opaque and semitransparent single-junction FAPbI3-based devices. (d) Trap density obtained from the SCLC method, (e) light intensity dependence of the *V*_OC_, and (f) normalized transient photovoltage decay of semitransparent FAPbI_3_-based devices.

We perform time-resolved photoluminescence (TRPL) and photoluminescence quantum yield (PLQY) measurements on stacks (i) and (ii). The stack (i) processed with both VAG and AS shows a mono-exponential decay in TRPL (Fig. 4(a)). VAG process exhibits a slightly increased charge-carrier lifetime (*τ* = 3581 ns) compared to AS method (*τ =* 3313 ns), which can be attributed to the improved grain size with fewer grain boundaries and free pinholes in VAG-FAPbI_3_ thin film (Fig. 3(b)) and pure-α-phase (Fig. 3(c) and (e)).^6,28,48^ The comparable average values of PLQY and implied open-circuit voltage (*V*_OC-imp_)^47,49^ (VAG: 2.5 × 10^−2^, 1.135 V; AS: 2.0 × 10^−2^, 1.129 V; see Fig. 4(b)) indicate negligible non-radiative recombination loss from the bulk and 2PACz/FAPbI_3_ interface. Stack (ii) shows a biexponential decay with a fast (*τ*_1_) and a slow lifetime (*τ*_2_) in TRPL (Fig. 4(a), Table S1, ESI†). The minimal values of *τ*_1_ (AS: 3 s; VAG: 4 s) in both fabrication methods reveal a very fast charge transfer from FAPbI_3_ to C_60_/SnO_*x*_ (Table S1, ESI†). Compared to AS method, a longer *τ*_2_ for VAG process (27 ns *vs.* 16 ns, Fig. 4(a)) demonstrates less non-radiative recombination at FAPbI_3_ and C_60_/SnO_*x*_ interface.^6,28,48^ We realize the main *V*_OC_ loss is related to the interface of FAPbI_3_ and C_60_/SnO_*x*_ since the PLQY decreases in the stack (ii) (VAG: from 2.5 × 10^−2^ to 1.2 × 10^−3^; AS: from 2.0 × 10^−2^ to 3.9 × 10^−5^, Fig. 4(b)). The corresponding average values of *V*_OC-imp_ decrease to 1.056 V for VAG process and 0.969 V for AS process, respectively. Importantly, the *V*_OC-imp_ loss at FAPbI_3_ and C_60_/SnO_*x*_ interface is reduced to 0.079 V for the VAG process, compared to that of 0.160 V for AS method (Fig. 4(b)). This matches well with the difference in *V*_OC_ (VAG: 1.08 V; AS: 1.00 V, Fig. S11a, ESI†). In addition, we find ALD temperature (90 °C for ∼150 min) does not affect the quality of FAPbI_3_ thin films as the PLYQ and *V*_OC-imp_ slightly increased after the samples of ITO/2PACz/FAPbI_3_ were heated up at the same condition (Fig. 4(b)).

We propose that a high trap density could originate from the defects at the interface of FAPbI_3_ and C_60_/SnO_*x*_ due to poor perovskite film quality and unstable α phase, thus resulting in a substantial trap-assisted non-radiative recombination.^3,50^ We utilize electroluminescence (EL) imaging on opaque and semitransparent devices to evaluate the defect distribution and film homogeneity. The EL signal increases with the local junction voltage, so the dark regions show local defects.^7,51,52^ As shown in Fig. 4(c), the devices processed with the VAG method present good homogeneity with significantly fewer defects. In contrast, the devices fabricated by the AS method show many local defects. In combination with the analyses of TRPL and PLQY (see Fig. 4(a) and (b)), we hypothesize these defects may mainly generate from the interfacial pinholes, cracks, inactive δ-phase, or wrinkles (*e.g.*, different local defect density in the hill or valley^29^) after the deposition of ETL. We further estimate the trap density (*n*_t_) employing space-charge-limited-current (SCLC) method.^6,28,53–55^ The *n*_t_ (Fig. 4(d) and Fig. S20a, ESI†) for VAG device (5.32 × 10^15^ cm^−3^) is less than a half compared to AS devices (11.22 × 10^15^ cm^−3^), demonstrating significantly reduced trap-assisted non-radiative recombination at the interface of FAPbI_3_ and C60/SnO_*x*_. We further investigate semitransparent devices utilizing light intensity dependence of the *V*_OC_ and transient photovoltage (TPV) characterizations. A lower ideality factor (*n*_id_; VAG: 1.12; AS: 1.44; Fig. 4(e)) and longer charge-recombination lifetime (VAG: 4.52 μs; AS: 2.63 μs; Fig. 4(f)) indicate that the charge recombination pathways in VAG device are efficiently blocked.^56,57^ This conclusion is further supported by reduced dark saturation current density (Fig. S20b, ESI†), a higher flat-band potential in Mott–Schottky plots (Fig. S20c, ESI†), and a higher charge recombination resistance in electrochemical impedance spectroscopy measurements (Fig. S20d and Table S2, ESI†), as they are in line with the data of TRPL, PLQY, SCLC, *n*_id_, and TPV (Fig. 4).

Overall, we demonstrate high-performance middle sub-cell by applying high-quality MBG FAPbI_3_ thin film is the key advance on triple-junction perovskite–perovskite–silicon solar cells. Compared to the optimum simulated *V*_OC_ (2.95 V, see simulation in Experimental procedures), there is still room to further reduce the *V*_OC_ loss in middle and top perovskite sub-cells.

### Durability of perovskite–perovskite–Si MTJSCs

**Fig. 5:**
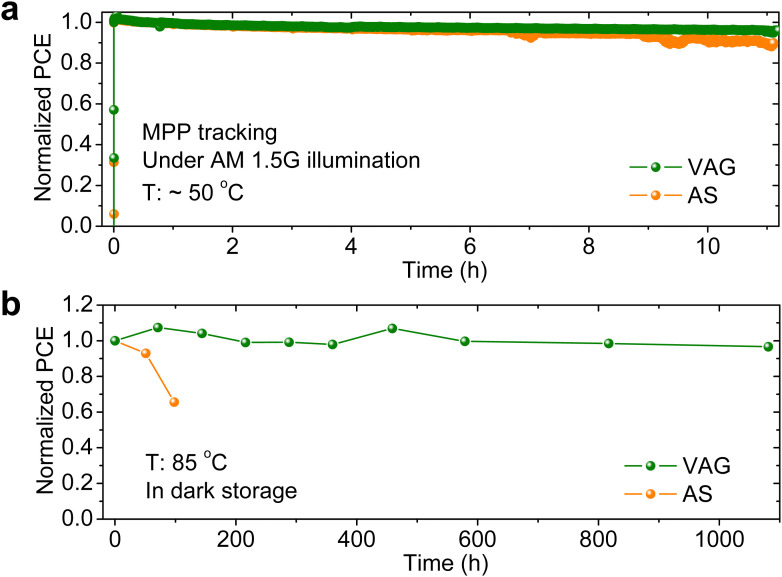
Durability of perovskite–perovskite–Si MTJSCs. Evolution of normalized PCEs of perovskite–perovskite–Si MTJSCs (a) under continuous AM 1.5G illumination at ∼50 °C for MPP tracking and (b) in dark storage at 85 °C. All devices without encapsulation were tested in an N_2_-filled glovebox.

Eventually, we evaluate the durability of our non-encapsulated perovskite–perovskite–Si MTJSCs in a N_2_ atmosphere. The stabilities of AS and VAG devices are first examined with MPP tracking under continuous AM 1.5G illumination (100 mW cm^−2^) at ∼50 °C (Fig. 5(a)). After 11 h, the VAG device retains 96% of initial PCE, which is more stable than AS device retaining 89% of the initial PCE. The slight drop in PCE of the champion triple-junction cell (with VAG-FAPbI_3_) during a long-term illumination (Fig. S21a, ESI†) might be triggered by light-induced phase segregation in the 1.84 eV sub-cell with high Br content (Fig. S21b, ESI†).^15,17,58^ We attribute the instability of the reference triple-junction cell (based on AS-FAPbI_3_) to phase segregation of WBG in the top cell (Fig. S21b, ESI†) and unstable MBG AS-FAPbI_3_ thin film in middle cell (Fig. S21c, ESI†). We further evaluate the thermal stability at an elevated temperature of 85 °C (Fig. 5(b)) in the dark. It is noteworthy the VAG device retains 96.6% of initial PCE after 1081 h, which is attributed to the superior thermal stability of VAG-FAPbI_3_ demonstrated in both opaque and semitransparent single-junction middle cells (Fig. S21d, ESI†). In contrast, the PCE of the AS device drops to 65.6% after 98 h, which is attributed to the imperfect AS-FAPbI_3_ film morphology (Fig. 3(a) and (b)) and α-to-δ phase transition (Fig. 3(c), (e) and Fig. S11a and S21d ESI†) that has been demonstrated in our previous work.^36^ These results indicate that the VAG process enables high-quality and stable middle perovskite thin film and thus offers a promising route to enhance long-term thermal stability in a triple-junction architecture. Future work needs to address the light stability of WBG perovskite solar cells by suppressing phase segregation.^18,19^ Different stress factors, *i.e.*, light, heat, bias, and humidity, *etc.* are necessary to evaluate the stability, which is key for the future commercialization of perovskite-based multi-junction PVs.^59^

## Conclusions

Using experimental optimizations and optical simulations, we successfully demonstrate high-efficiency perovskite–perovskite–Si MTJSCs, achieving a record PCE of 24.4%. A high overall *J*_SC_ is realized up to 11.6 mA cm^−2^ by optimized light management and good current matching for both perovskite sub-cells. Using an AS-free VAG process enables a high-quality FAPbI_3_ middle perovskite sub-cell (free of wrinkles, cracks, and pinholes), resulting in low non-radiative recombination loss at the perovskite/ETL interfaces. This improves *V*_OC_ up to 2.84 V in a triple-junction architecture. Non-encapsulated MTJSCs retain 96.6% of initial PCE in dark storage aging at 85 °C for 1081 h. Our work offers advanced approaches for the fabrication of efficient perovskite-based triple-junction PVs.

## Experimental procedures

### Materials

Lead iodide (PbI_2_, TCI, 99.99%), lead bromide (PbBr_2_, TCI), formamidinium iodide (FAI, GreatCell Solar), formamidinium bromide (FAI, GreatCell Solar), methylammonium chloride (MACl, Dyenamo), cesium iodide (CsI, Alfa Aesar), cesium bromide (CsBr, Alfa Aesar), [2-(9*H*-carbazol-9-yl)ethyl] phosphonic acid (2PACz, TCI), fullerene (C_60_, Sigma Aldrich, 99.5%), 2,9-dimethyl-4,7-diphenyl-1,10-phenanthroline (BCP, Lumescence Technology), lithium fluoride (LiF, Luminescence Technology), magnesium fluoride (MgF_2_, Sigma Aldrich), dimethylformamide (DMF, Sigma Aldrich, anhydrous, 99.8%), dimethyl sulfoxide (DMSO, Sigma Aldrich, anhydrous, ≥99.9%), ethyl ethanoate (EA, Sigma Aldrich, anhydrous, 99.8%), ethanol (VWR Chemicals, absolute, 99.8%), tetrakis(dimethylamino)tin(iv) (TDMASn, 99.99%-Sn, Strem Chemicals), indium tin oxide (ITO) or indium zinc oxide (IZO), hydrogen-doped indium oxide (IOH) (using InO/ZnO target, Kurt J. Lesker Company, 90/10 wt%, 99.99%), nickel oxide (NiO_*x*_) (using NiO_*x*_ target, Kurt J. Lesker Company, 99.995%).

### Preparation of middle perovskite (∼1.52 eV) precursor

An optimum 1.3 M FAPbI_3_ perovskite precursor was prepared in a N_2_-filled glovebox (O_2_ < 0.5 ppm and H_2_O < 0.4 ppm) from 1.43 M PbI_2_, 1.3 M FAI, and 30 mol% MACl dissolved in a mixed solvent of DMF and DMSO with a volume ratio of 4 : 1. The thickness of MBG perovskite layer was optimized by varying the concentration of perovskite precursor from 1.1 to 1.9 M.

### Preparation of top perovskite (∼1.84 eV) precursor

An optimum 0.8 M FA_0.8_Cs_0.2_Pb(I_0.5_Br_0.5_)_3_ perovskite precursor was prepared in a glovebox from 0.4 M PbI_2_, 0.4 M PbBr_2_, 0.32 M FAI, 0.32 M FAI, 0.08 M CsI, and 0.08 M CsBr dissolved in a mixed solvent of DMF and DMSO with a volume ratio of 4 : 1. The thickness of WBG perovskite layer was optimized by varying the concentration of perovskite precursor from 0.6 to 1.0 M.

### Deposition of middle perovskite (∼1.52 eV) thin film

FAPbI_3_ perovskite thin film was fabricated using either anti-solvent (AS) quenching or vacuum-assisted growth (VAG) methods.^6,7^ For the AS process, the FAPbI_3_ solution was deposited by spin-coating at 1000 rpm for 10 s and 5000 rpm for 40 s. 150 μL EA was dropped at 30 s during the second step of spin coating. For the VAG process, the FAPbI_3_ solution was deposited by spin-coating at 5000 rpm for 30 s, and the thin film was promptly transferred into a vacuum chamber with an optimized vacuum time of 30 s. Wet FAPbI_3_ thin films fabricated from both methods were then annealed at 120 °C for 20 min.

### Deposition of top perovskite (∼1.84 eV) thin film

FA_0.8_Cs_0.2_Pb(I_0.5_Br_0.5_)_3_ perovskite thin film was deposited by spin-coating at 1000 rpm for 10 s and 5000 rpm for 30 s. 150 μL EA was dropped at 11 s during the second step of spin coating. The wet FA_0.8_Cs_0.2_Pb(I_0.5_Br_0.5_)_3_ thin film was then annealed at 150 °C for 20 min.

### Atomic layer deposition of tin oxide (SnO_*x*_) layer

A 35-nm SnO_*x*_ thin film was fabricated by atomic layer deposition (ALD) with 300 cycles in an ALD system (Picosun, R200 Advanced) at 90 °C using the precursors of TDMASn (pulse time 1.6 s, purge time 12 s) and water (pulse time 0.1 s, purge time 16 s), as reported in our previous work.^7^ High-purity argon (Ar, 99.999%) was used as carrier gas and purge gas. The line flows of TDMASn and water were set to 120 and 150 standard cubic centimeters per minute (sccm), respectively. The TDMASn source container was preheated for 1 hour at 70 °C to ensure thermal equilibrium.

### Fabrication of single-junction perovskite (∼1.52 eV or ∼1.84 eV) solar cells

The single-junction perovskite solar cells (middle or top sub-cell) were fabricated in a p–i–n architecture of ITO/2PACz/perovskite/(LiF)/C_60_/BCP/gold (Au). ITO substrates (sheet resistance 15 Ω sq^−1^, Luminescence Technology) were cleaned with acetone and isopropanol in an ultrasonic bath for 10 min, respectively. Substrates were further treated with an oxygen plasma for 3 min. A ∼0.475 mg mL^−1^ 2PACz (in anhydrous ethanol) solution was spin-coated on the ITO substrates at 3000 rpm for 30 s and subsequently annealed at 100 °C for 10 min. FAPbI_3_ perovskite thin films were fabricated using VAG and AS methods. FA_0.8_Cs_0.2_Pb(I_0.5_Br_0.5_)_3_ perovskite thin film was fabricated with AS method, and a 1-nm LiF thin layer was subsequently thermally evaporated on top. Finally, 30-nm C_60_, 5-nm BCP, and 75-nm Au were thermally evaporated to complete the devices. The active area of single-junction devices was 0.105 cm^2^.

### Fabrication of silicon bottom cells

The silicon (Si) bottom cells were fabricated from polished n-type Float Zone (FZ) wafers (∼2 Ω cm) with a thickness of ∼200 μm after potassium hydroxide (KOH) etching before the fabrication process. A thick silicon dioxide (SiO_*x*_) was grown to isolate the rear minority carrier hole contact from the defective cleaved edge of the finished cell. The cell's active area was defined by ablating the SiO_*x*_ locally from the rear side and KOH etching to remove any laser damage. The SiO_*x*_ from the front side was removed by a single-side treatment with hydrofluoric (HF) acid. We grew a thin SiO_*x*_ and deposited amorphous Si (a-Si) on top of the SiO_*x*_. We doped the a-Si layer by implanting pH and Boron to the front and rear sides, respectively, and formed the electron- and hole-selective passivating polysilicon-on-passivating-oxide (*n*-POLO and *p*-POLO) contacts by furnace annealing and oxidation. The grown SiO_*x*_ on the rear side was patterned by laser ablation such that only islands of *p*-POLO contacts remained at the rear side after KOH etching and texturization. The SiO_*x*_ protecting the front *n*-POLO contact was removed by single-side HF treatment, and the front contact's poly-Si was thinned to ∼50 nm in an ammonium–peroxide mixture. The rear side was passivated by a stack of aluminum oxide (Al_2_O_3_), silicon nitride (SiN_y_), and SiOx, and the front side received an Al_2_O_3_ layer for hydrogenation of the *n*-POLO front contact. The dielectric layer stack on the rear side was ablated locally on the *p*-POLO islands to provide electrical contact to the metal. Next, the cell precursor was dipped in diluted HF to remove the Al_2_O_3_ hydrogenation source from the front side, and 20 nm ITO was sputtered on the front *n*-POLO contact. Finally, the aluminum (Al) back contact was evaporated on the rear side, and 16 × 16 mm^2^ substrates with 1 cm^2^ cells were cleaved from the wafer.

### Fabrication of perovskite–perovskite–Si monolithic triple-junction solar cells

The architecture of monolithic triple-junction solar cells (MTJSCs) is Si/ITO/NiO_*x*_/2PACz/FAPbI_3_/C_60_/SnO_*x*_/ITO/NiO_*x*_/2PACz/Cs_0.2_FA_0.8_Pb(I_0.5_Br_0.5_)_3_/LiF/C_60_/SnO_*x*_/IZO/Au/MgF_2_. Si substrates with ∼20 nm ITO layer on the front side were cleaned with isopropanol by spin-coating. A ∼15-nm NiO_*x*_ layer was sputtered on top of Si/ITO substrates. 2PACz, FAPbI_3_, Cs_0.2_FA_0.8_Pb(I_0.5_Br_0.5_)_3_, and LiF thin films were fabricated in the same condition as with the single-junction device. C_60_ thin films were thermally evaporated with thicknesses of ∼15 nm. A 35-nm SnO_*x*_ layer was fabricated by ALD. The middle ITO recombination layer (∼15 nm) and top IZO electrode were fabricated by a sputtering system. A ∼300-nm Au was then thermally evaporated with a c-shaped mask (framing an active area of 52.25 mm^2^). Finally, a ∼125-nm MgF_2_ antireflection coating was thermally evaporated to complete the triple junction.

### Current-density–voltage (*J*–*V*) and maximum power point (MPP) tracking measurements

The *J*–*V* characteristics of the devices were measured using a class AAA 21-channel LED solar simulator (Wavelabs Solar Metrology Systems Sinus-70) equipped with a source meter (Keithley 2400) with an air-mass AM 1.5G spectrum (100 mW cm^−2^ illumination). The scan rate was set to 0.6 V s^−1^, and a certified Si reference solar cell (KG0, Newport) was used for calibration of the illumination intensity of the solar simulator. The stabilized power conversion efficiency (SPCE) was determined by maximum power point (MPP) tracking under continuous AM 1.5G illumination. The temperature of the devices was not controlled during *J*–*V* measurements. Devices were placed on a hotplate at 85 °C in the dark for thermal stability testing.

### External quantum efficiency (EQE) measurements

EQE spectra for triple-junction solar cells were measured using a Bentham PVE300 system with a modulated monochromatic light. A chopping frequency was set to ∼580 Hz, and an integration time was set to 500 ms. The EQE response was calibrated using Si and germanium (Ge) certified reference cells for 300–1100 nm and 1000–1300 nm wavelength regions, respectively. For the EQE measurements of each sub-cell, we used a combination of different filters and bias light sources to saturate the other two sub-cells. For the top cell, the light-emitting diode (LED) lights (780 nm and 940 nm) and a long-pass filter (with cut-on wavelength at 850 nm, FGL850M) were used. For the middle cell, the LED lights (465 nm and 940 nm) and a long-pass filter (FGL850M) were used. For the bottom cell, the LED lights (465 nm and 780 nm) and a bandpass filter (335–610 nm, FGB37M) were used.

### Scanning electron microscopy (SEM)

Top-view and cross-sectional SEM images of the perovskite thin films and triple-junction solar cells were taken using a Zeiss Supra60 VP SEM system.

### Atomic force microscopy (AFM)

Surface topographies of perovskite thin films were inspected using Nano Wizard II (JPK Instruments). The scanning area was 10 μm × 10 μm.

### Optical microscopy

Optical microscopy images of perovskite thin films were collected using ZEISS Axioplan 2 microscope.

### X-ray diffraction (XRD)

XRD was performed on the layer stack of ITO/2PACz/FAPbI_3_ using a Bruker D2Phaser system with Cu-Kα radiation (*λ* = 1.5405 Å) in Bragg–Brentano configuration using a LynxEye detector.

### Time-resolved photoluminescence (TRPL)

TRPL was performed in ambient air based on an FLSP920 Fluorescence Spectrometer (Edinburgh Instruments Ltd.) using the TCSPC acquisition technique. A picosecond pulsed laser diode (PicoQuant, 635 nm) externally triggered by a delay generator (repetition rate: 500 kHz) was used as the excitation light. The emission was collected by a photomultiplier tube (Hamamatsu R928P).

### Photoluminescence quantum yield (PLQY)

PLQY measurements were conducted inside an integrating sphere (LabSphere, 15 cm diameter) in ambient air. A green laser (Coherent or LD-515-10MG from Roithner Lasertechnik) was directed into the sphere *via* a small entrance port. An optical fiber was used to collect the emission from the exit port of the sphere and guide it to the spectrometers (QE65 Pro from Ocean Optics and AvaSpec-ULS2048x64TEC from Avantes). Spectral response was calibrated using a calibration lamp (HL-3plus-INT-Cal from Ocean Optics). Raw measured spectra were recalculated to give power spectra using the integration time. The samples were placed at an angle of 15° with respect to the laser beam to avoid specular reflectance toward the entrance port. The ‘implied *V*_OC_’ was derived *via*:^46,47,60^
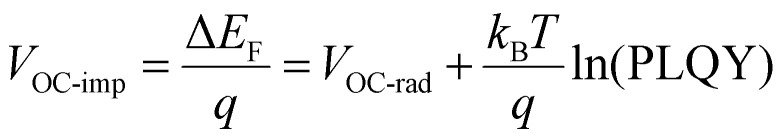


*V*_OC-rad_ be estimated by the following equation:^61^
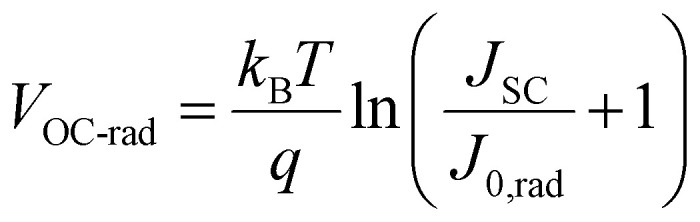
where *k*_B_ is the Boltzmann constant, *T* is the absolute temperature (300 K), *q* is the elementary charge, *J*_SC_ is the short-circuit current density, and *J*_0,rad_ is radiative saturation-current density. *J*_SC_ and *J*_0,rad_ can be estimated integrating the overlap of the EQE with AM 1.5G illumination of solar spectrum and black body spectrum at 300 K over the energy, respectively. *V*_OC-rad_ was calculated to be ∼1.23 eV.

### Mott–Schottky (MS) measurements

MS measurements for single-junction solar cells were performed using a PAIOS system (Fluxim AG) in the dark with a constant frequency of 30 kHz and an amplitude of 20 mV.

### Dark J–V curves and ideality factor measurements

Dark *J*–*V* curves and ideality factor measurements were performed using a PAIOS system with a white light emitting diode (Cree XP-G). *V*_OC_ can be estimated by the following equation:^6,62^
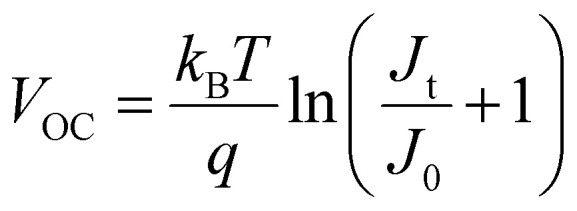
where *k*_B_ is the Boltzmann constant, *T* is the absolute temperature, *q* is the elementary charge, *J*_t_ is the theory of current density, and *J*_0_ is the reverse saturation current density.

The ideality factor was studied by measuring light intensity-dependent *V*_OC_. Logarithmic *V*_OC_ can be linear fitted by the equation:^6,63^
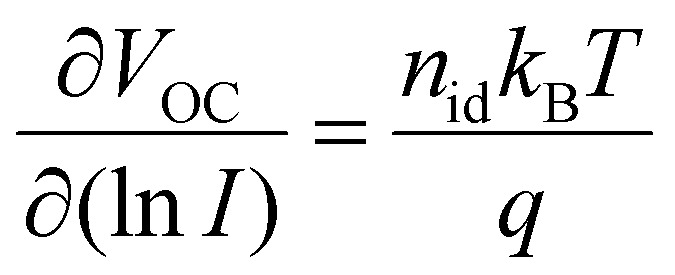
where *n*_id_ is the ideality factor, *k*_B_ is the Boltzmann constant, *T* is the absolute temperature, *q* is the elementary charge, and *I* is the light intensity. The *n*_id_ values are reflected in the curve slopes.

### Space-charge-limited current (SCLC) measurements

SCLC measurements were conducted on the electron-only single-junction semitransparent devices in the architecture of ITO/SnO_2_/FAPbI_3_/C_60_/SnO_*x*_/IZO/Au. Dark current-density*–*voltage (*J*–*V*) characteristics were performed using a PAIOS system in the dark at the range of 0*–*2 V with a settling time of 40 ms. The trap density (*n*_t_) can be expressed in the following equation:^6,28,53–55,64^
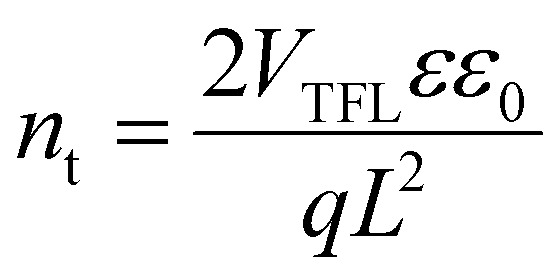
where *V*_TFL_ is the trap-filling limit voltage, *L* is the thickness of the perovskite thin film, *ε* is the relative dielectric constant of perovskite, *ε*_0_ is the vacuum permittivity, and *q* is the elementary charge.

### Electrical impedance spectroscopy (EIS)

EIS was performed using PAIOS system equipped with a white light emitting diode (Cree XP-G) under a bias of 1 V and an amplitude of 30 mV. Nyquist plots were fitted by the Z-View program.

### Transient photovoltage (TPV) measurements

TPV measurements were conducted using a PAIOS system. A high resistor (1 MΩ) is used to form an open-circuit condition, so that the current is zero during the whole measurement. A small perturbation light pulse to the background illumination is applied to a constant offset light intensity.

### Electroluminescence (EL) and photoluminescence (PL) imaging

The EL and PL images were acquired with a 2.1 megapixel scientific CMOS camera (Quantalux sCMOS camera, Thorlabs). The EL and PL were filtered with a 775 nm shortpass (Edmund Optics) stacked on top of a 665nm longpass (Thorlabs) to remove the excitation light during PL imaging. Two blue LED bars (CCS Inc.) were used an excitation source for PL imaging. The perovskite thin films or solar cells were biased using a Keithley 2450 SMU, as it was reported in our previous work.^7^ All measurements were performed in ambient air.

### Simulations

Simulations were performed using the open-source modelling platform EYcalc.^65^ Details of this modeling platform have been published in our previous work.^5,34,35^ The optical simulations apply state-of-the-art optical data and electrical parameters that were derived from optical and electrical characterization of our single-junction devices.^35^ The thickness of each layer was chosen as follow: MgF_2_ (125 nm)/IZO (90 nm)/SnO_*x*_ (20 nm)/C_60_ (15 nm)/top perovskite (varied)/2PACz-NiO_*x*_ (5 nm)/ITO (15 nm)/SnO_*x*_ (20 nm)/C_60_ (15 nm)/middle perovskite (varied)/2PACz-NiO_*x*_ (15 nm)/ITO (5 nm)/a-Si(n) (40 nm)/a-Si(i) (10 nm)/c-Si (250 μm)/a-Si(i) (10 nm)/a-Si(p) (10 nm)/ITO (80 nm)/Ag (250 nm). The champion perovskite–perovskite–Si monolithic triple-junction solar cell was chosen using a 1.84 eV top perovskite with a thickness of 200 nm and 1.52 eV middle perovskite with a thickness of 1000 nm. The best simulated power conversion efficiency is 24.8% with an open-circuit voltage of 2.95 V and a FF of 0.717.

In this study, we use our in-house developed EY modelling platform EYCalc available as an open-source software project.^35^ A comprehensive description of the software is provided by Schmager *et al.*^35^ Here, we provide a brief overview of its structure and working principles. This platform consists of four modules: irradiance, optics, electrics, and EY module. Based on data from the typical meteorological year (TMY3)^66^ for various locations in the USA, the irradiance module computes direct and diffuse irradiance spectra for each hour of the year. Then we extract the appropriate hourly-resolved irradiance from the TMY3 data using a cloud model and the simplified model of atmospheric radiative transfer of sunshine (SMARTS).^67^ In analyses conducted under standard test conditions, the platform foregoes the utilization of location-specific irradiance spectra, opting instead to employ solely the AM 1.5G spectrum. Afterward, the optics module calculates spectrally and angularly resolved optical properties, including absorptance, reflectance, and transmittance for the provided layer stack. The optics module computes the optical parameters by combining a series expansion of Beer–Lambert law or the transfer matrix method (TMM), depending on whether the layer is optically coherent (thin) or optically incoherent (thick), respectively. The platform is able to handle textured interfaces using geometrical ray tracing.^68^

Next, we calculate the hourly resolved photogenerated current density (*J*_G_) in the absorber layers by the EY module by merging the output from the irradiance and optics modules while considering the solar cell's rotation and tilt. Then, we determine the photovoltaic parameters in the electrical module. The electrical module subsequently calculates temperature-dependent *J*–*V* characteristics and MPP for each hourly resolved *J*_G_. For this process, an analytical one-diode or two-diode model or a precise numerical method can be used. In this study, we used the second method by implementing a two-diode model in LTspice.^69^ The EY module then calculates the annual EY by considering each hour's contributions throughout the year for different climatic locations. In case of STC analysis, the PCE of the solar cell is computed based on the values obtained in the previous steps. Temperature effects are taken into account using temperature coefficients for the *V*_OC_ and *J*_G_. The cell temperature is estimated using the Nominal Operating Cell Temperature (NOCT) model, assuming NOCT = 48° and extracting the insolation on the cell and ambient air temperature from TMY3 data.

## Author contributions

H. H., B. A. N., and U. W. P. convinced the idea for this project. H. H. did the fabrication of triple-junction solar cells and the rest of characterizations. S. X. A., S. O., and F. G. contributed to the simulation of triple-junction solar cells. Y. L. conducted TRPL and PLQY measurements. R. S. conducted XRD. F. S. and F. L. conducted the EL imaging. Q. J. performed AFM. H. H. and T. F. fabricated ALD-SnO_*x*_ layers. B. A. N. and A. D. performed SEM. H. H. and T. P. optimized 2PACz layer. S. M. optimized sputtering TCO. M. R. and R. P. provided the Si bottom cells and contributed with their expertise on Si solar cells. B. A. N. and U. W. P. supervised the project. H. H. wrote the original draft. B. A. N. and U. W. P. reviewed and edited the manuscript. All authors contributed to the discussions and the final reviewing of the manuscript.

## Conflicts of interest

There are no conflicts to declare.

## Supplementary Material

EE-017-D3EE03687A-s001
