# Supplementary material for: Triple-junction perovskite–perovskite–silicon solar cells with power conversion efficiency of 24.4%
Source: Energy Environ Sci. 2024 Feb 13;17(8):2800–14. doi: 10.1039/d3ee03687a (PMC11036531; doi:10.1039/d3ee03687a)
Supplement: EE-017-D3EE03687A-s001 [file EE-017-D3EE03687A-s001.pdf]

Electronic Supplementary Material (ESI) for Energy & Environmental Science.
This journal is © The Royal Society of Chemistry 2024

Electronic Supplementary Information

Triple-junction perovskite–perovskite–silicon solar cells with power conversion efficiency of 24.4%

Hang Hu,[ab] Sophie X. An,[b] Yang Li,[ab] Seyedamir Orooji,[ab] Roja Singh,[ab] Fabian Schackmar,[ab] Felix Laufer,[ab] Qihao Jin,[b] Thomas Feeney,[ab] Alexander Diercks,[b] Fabrizio Gota,[ab] Somayeh Moghadamzadeh,[ab] Ting Pan,[ab] Michael Rienäcker,[c] Robby Peibst,[cd] Bahram Abdollahi Nejand*[ab] and Ulrich W. Paetzold*[ab]

[a] Institute of Microstructure Technology (IMT), Karlsruhe Institute of Technology (KIT), Hermann-von-Helmholtz-Platz 1, 76344 Eggenstein-Leopoldshafen, Germany

[b] Light Technology Institute (LTI), Karlsruhe Institute of Technology (KIT), Engesserstrasse 13, 76131 Karlsruhe, Germany

[c] Institute for Solar Energy Research Hamelin (ISFH), Am Ohrberg 1, 31860, Emmerthal, Germany

[d] Institute of Electronic Materials and Devices, Leibniz Universität Hannover, Schneiderberg 32, 30167, Hannover, Germany

* Corresponding authors: bahram.abdollahi@kit.edu; ulrich.paetzold@kit.edu

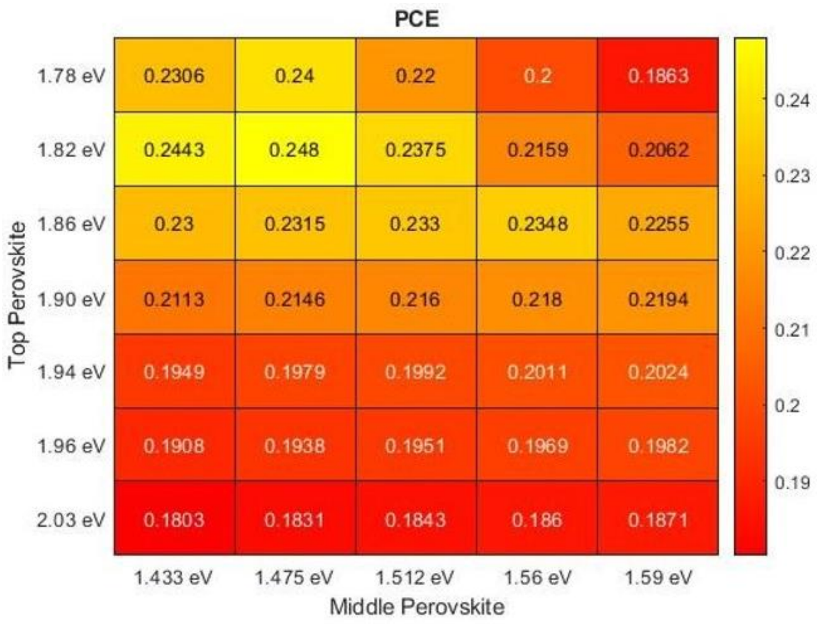

Fig. S1 Heatmap of simulated PCEs of perovskite–perovskite–Si MTJSCs for varied bandgap combinations of top and middle perovskites.

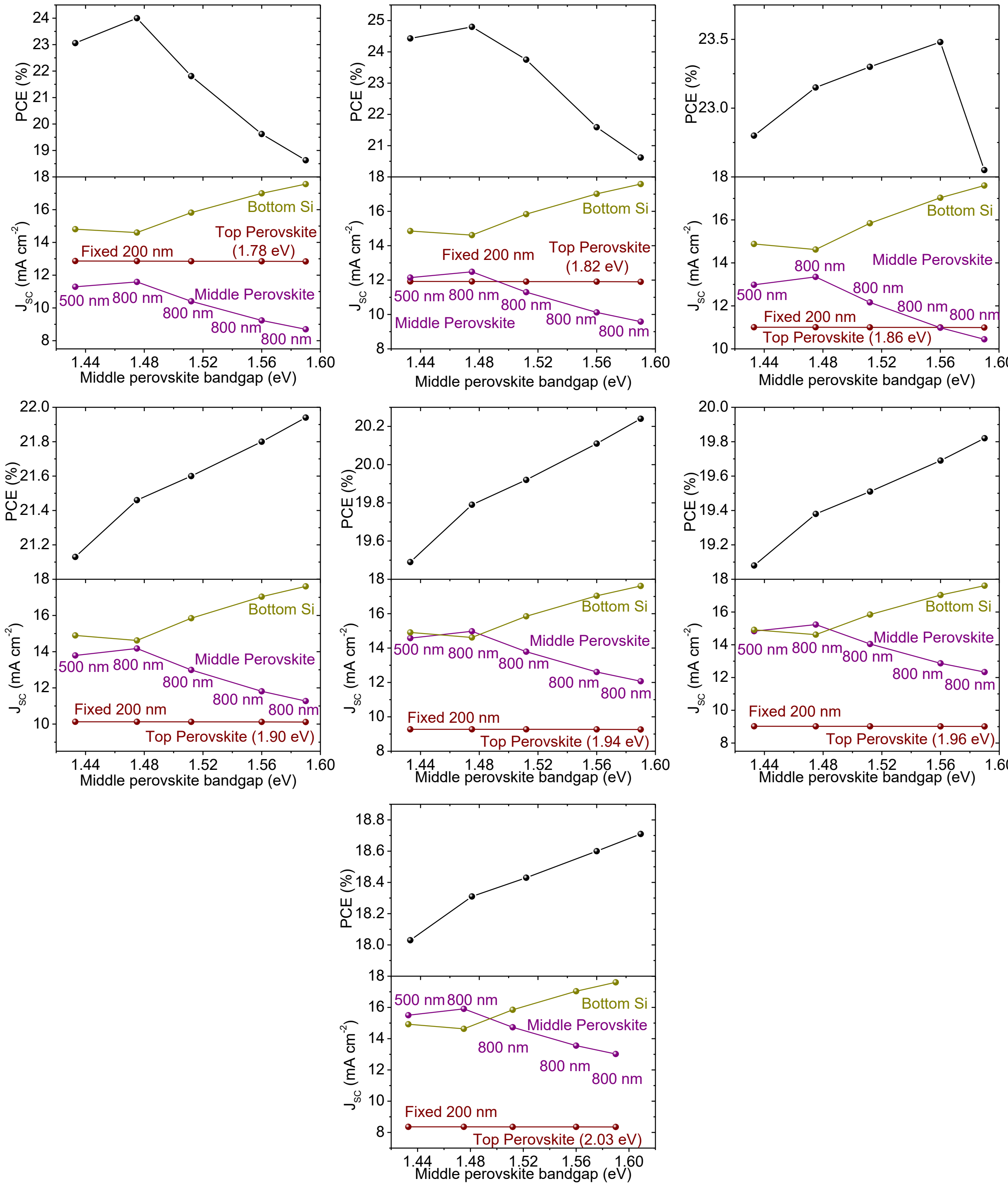

Fig. S2 Simulated data of PCE and J_{SC} of perovskite–perovskite–Si MTJSCs for varied bandgap combinations of top and middle perovskites. The thickness of top perovskite is fixed to 200 nm.

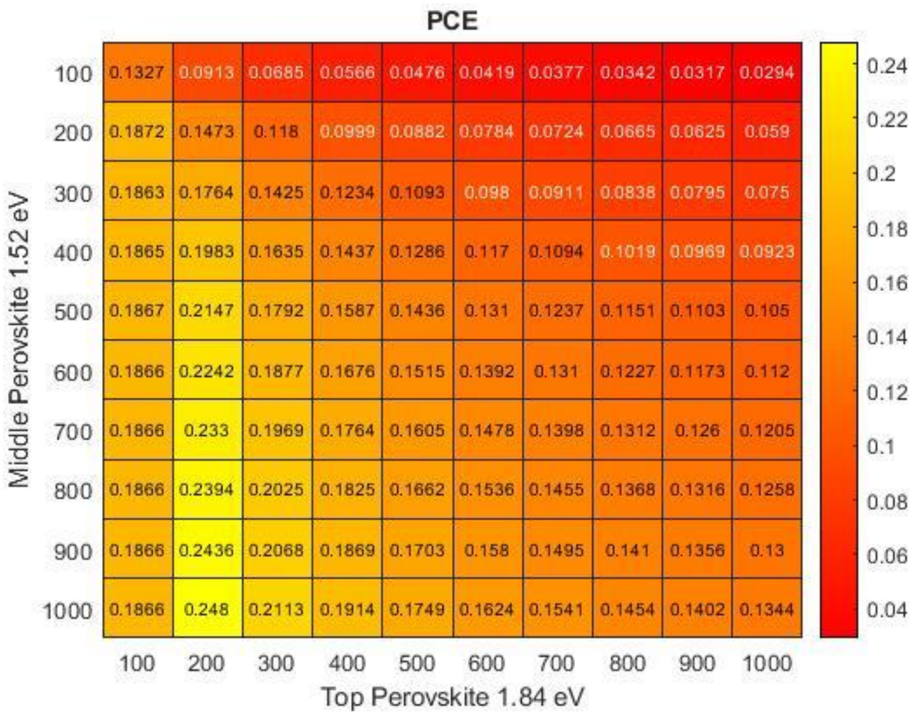

Fig. S3 Heatmap of simulated PCEs of perovskite–perovskite–Si MTJSCs for varied thickness of top (1.84 eV) and middle (1.52 eV) perovskites.

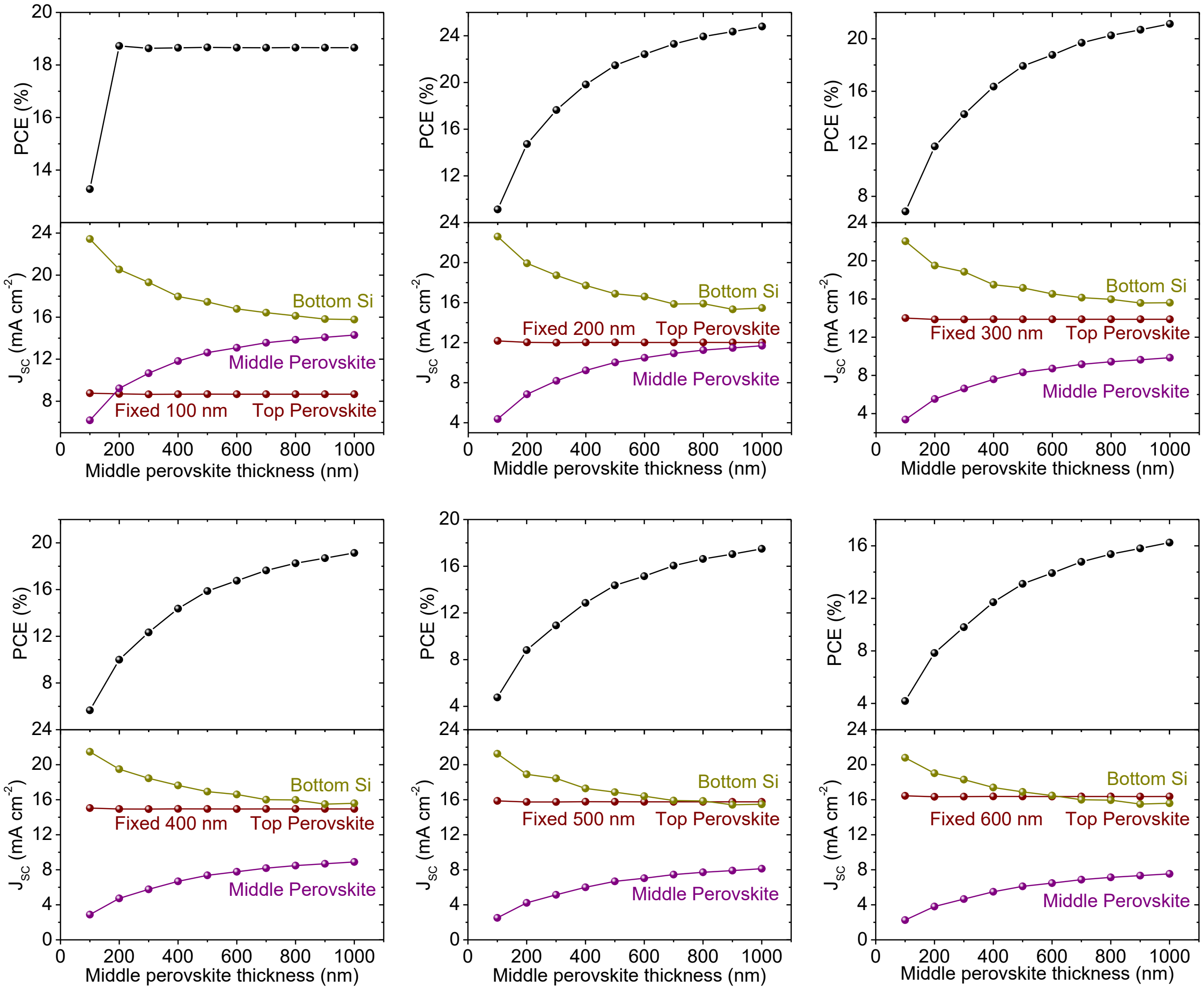

Fig. S4 Simulated data of PCE and J_{SC} of perovskite–perovskite–Si MTJSCs for varied thickness of top (1.84 eV) and middle (1.52 eV) perovskites.

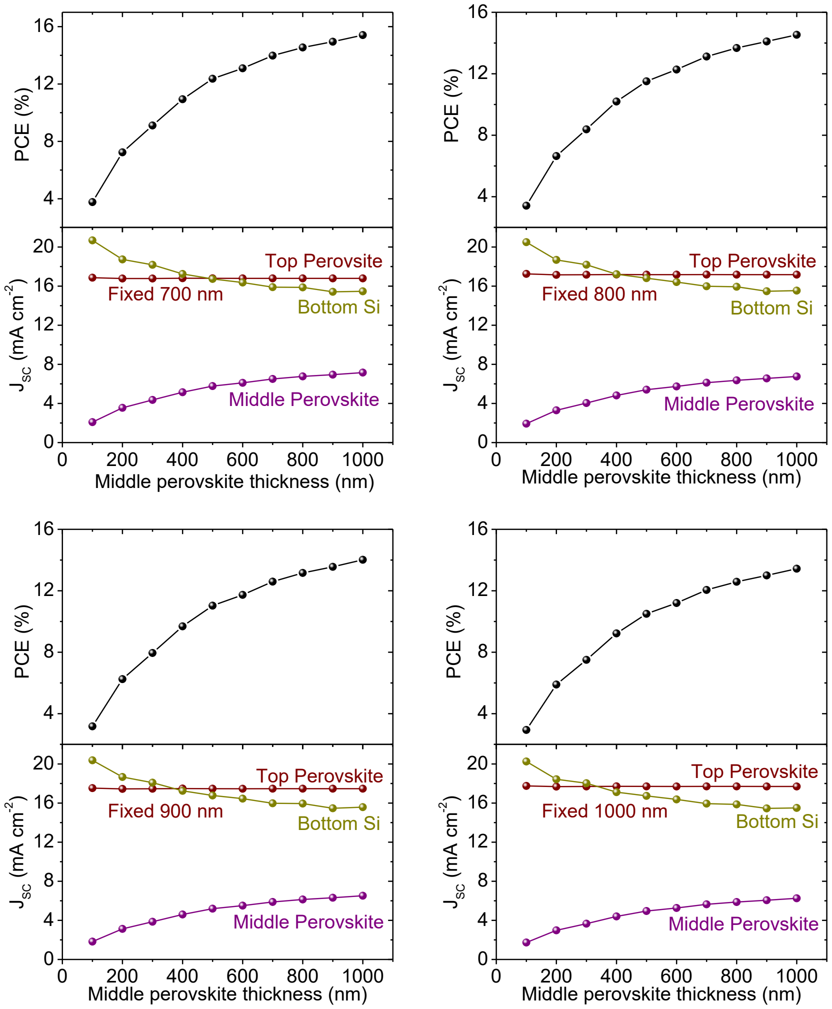

Fig. S5 Simulated data of PCE and J_{SC} of perovskite–perovskite–Si MTJSCs for varied thickness of top (1.84 eV) and middle (1.52 eV) perovskites.

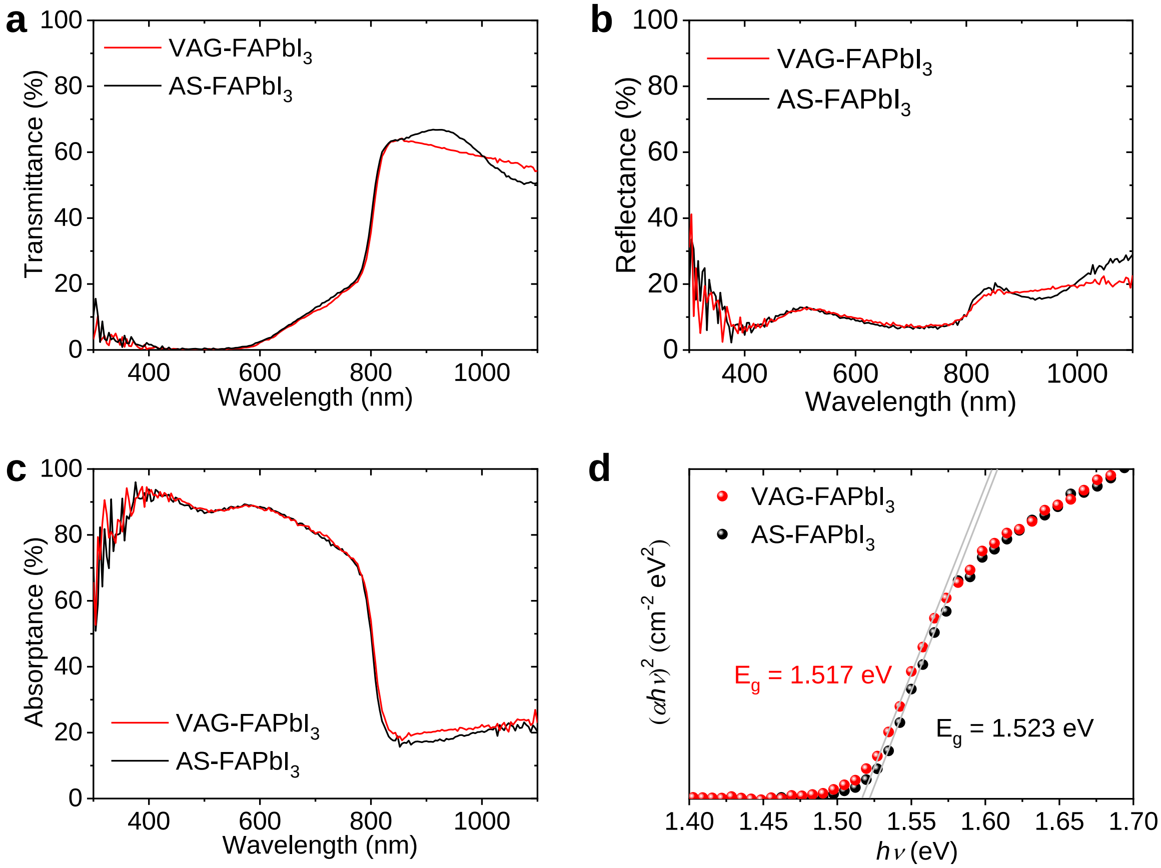

Fig. S6 (a) Transmittance, (b) reflectance, (c) absorptance spectra, and (d) Tauc-plots for $FAPbI_3$ perovskite thin films fabricated by AS and VAG methods.

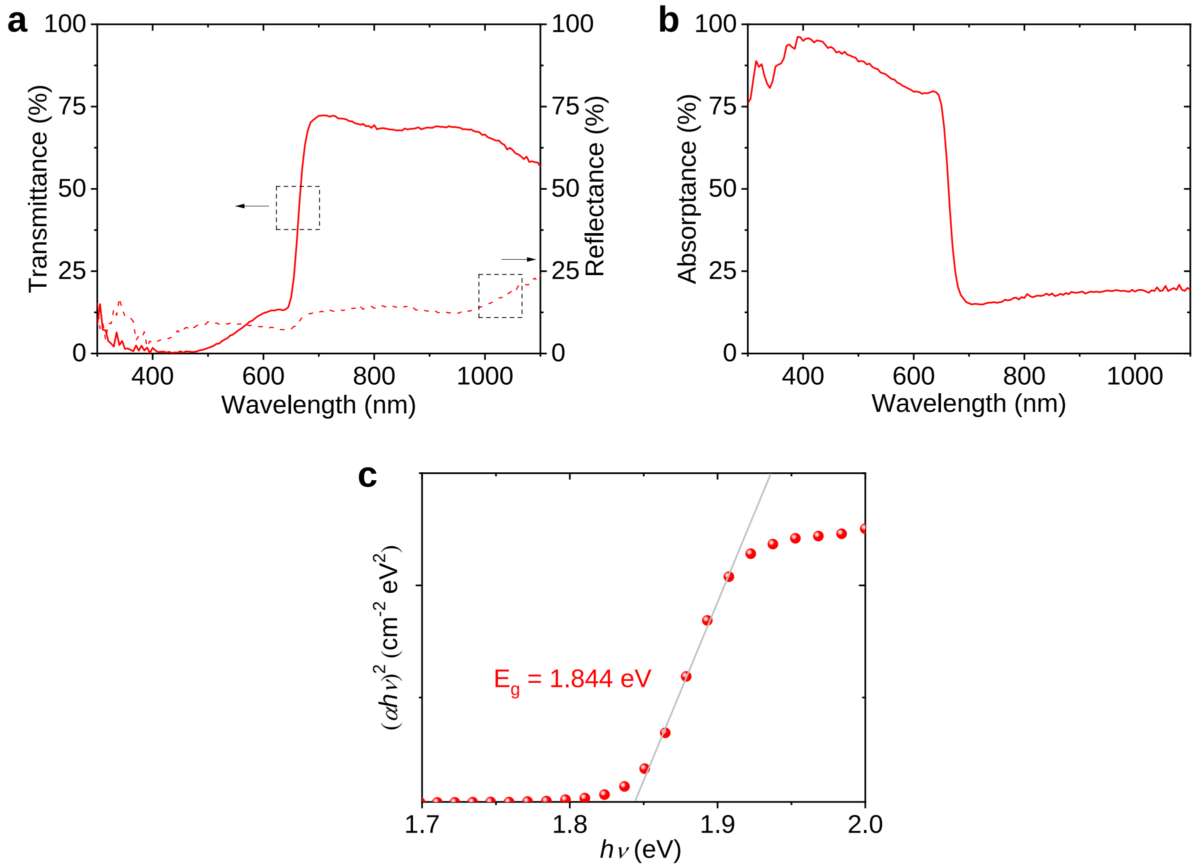

Fig. S7 (a) Transmittance, reflectance, (b) absorptance spectra, and (c) Tauc-plots for WBG perovskite thin film fabricated by AS method.

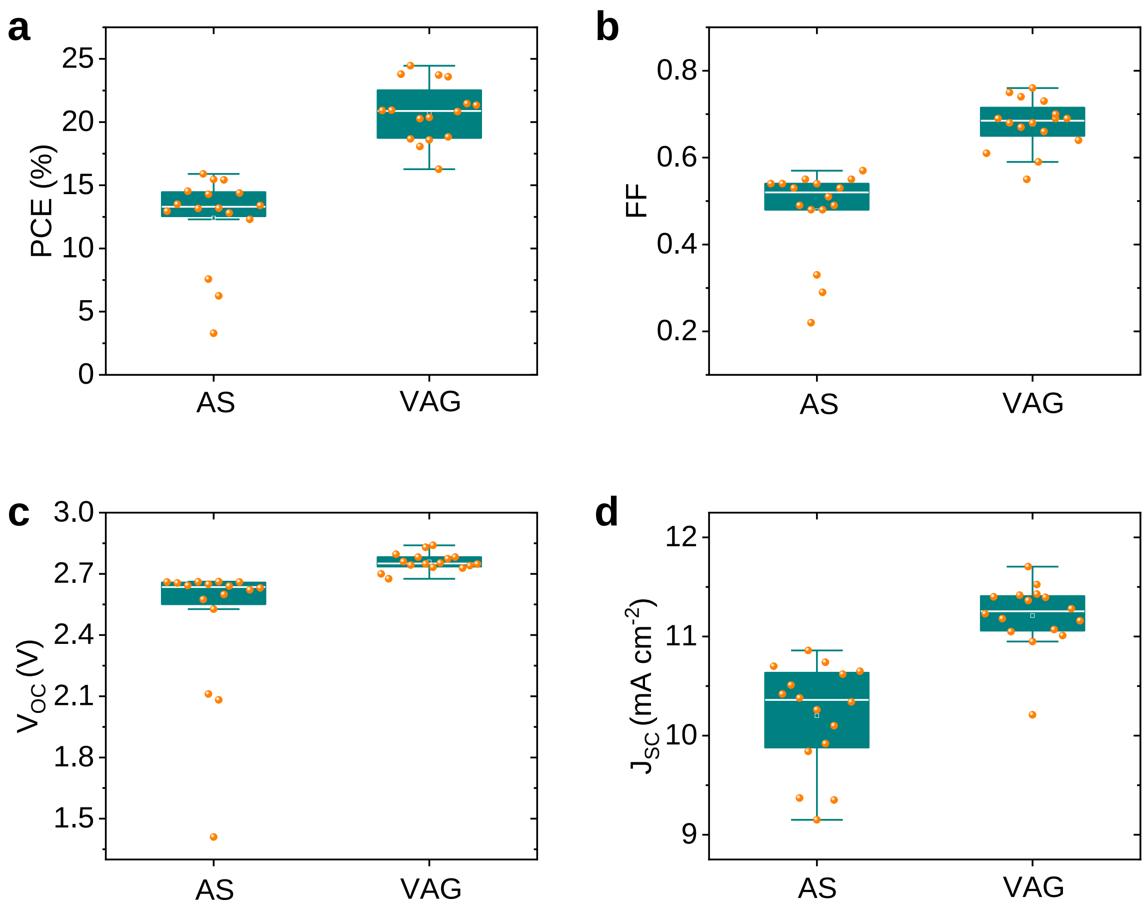

Fig. S8 Statistical distribution of the photovoltaic parameters for perovskite–perovskite–Si MTJSCs. (a) power conversion efficiencies (PCE), (b) fill factor (FF), (c) open-circuit voltage (V_{OC}), and (d) short-circuit current density (J_{SC}). The MBG $FAPbI_3$ thin films of middle cells were fabricated by AS and VAG methods.

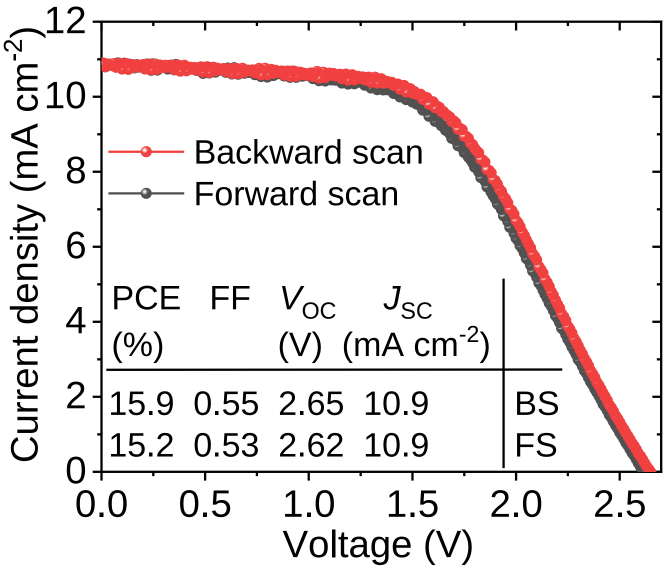

Fig. S9 *J–V* characteristics for the champion perovskite–perovskite–Si MTJSC based on AS method for processing of MBG $FAPbI_3$ perovskite thin film.

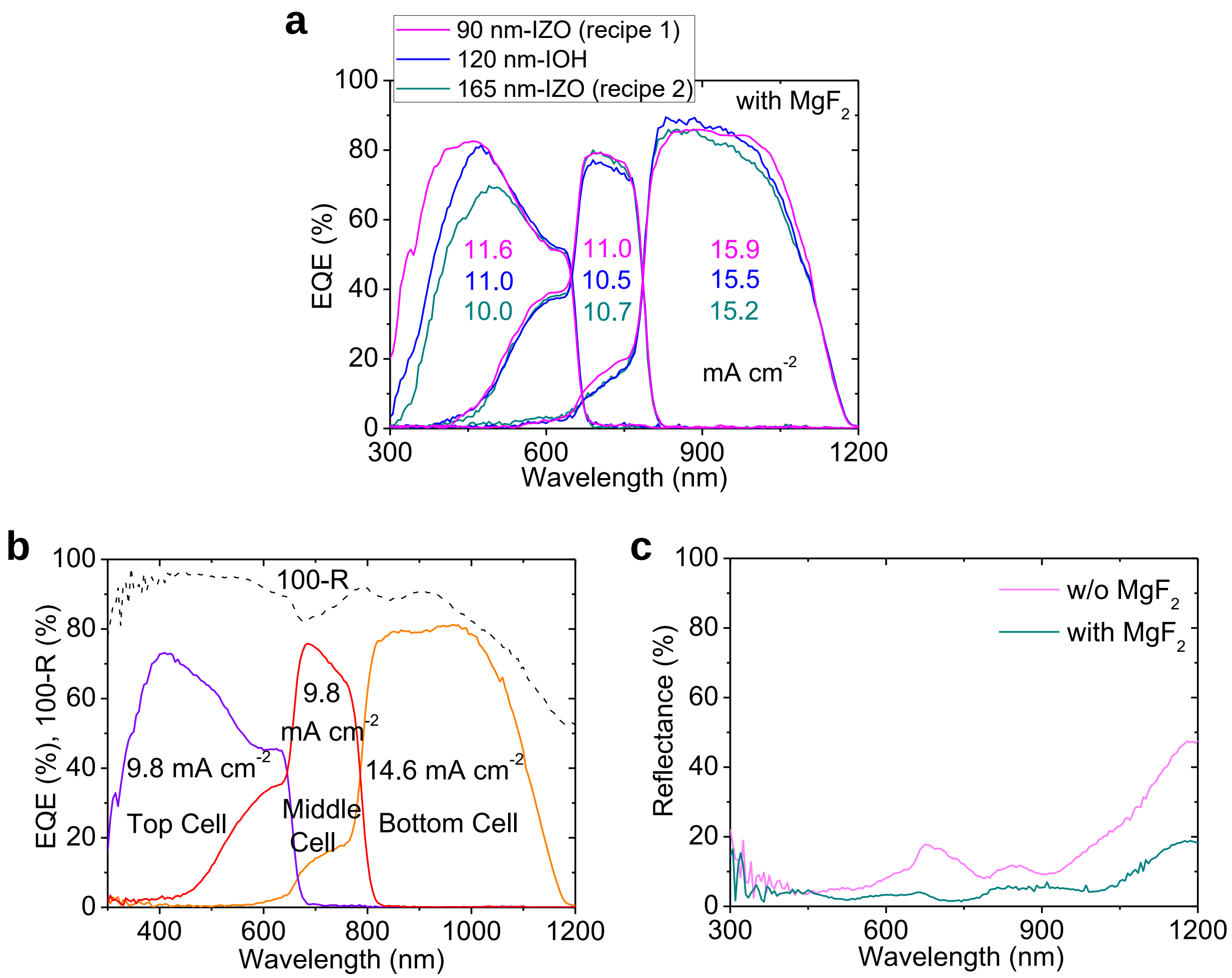

Fig. S10 EQE and reflectance spectra for the MTJSC combined with perovskite bandgaps of 1.84 eV and 1.52 eV. (a) Using different TCO layers (recipe 1: 90 nm IZO with a low deposition pressure of 0.8 mTorr and a high power supply of 200 W; recipe 2:165 nm IZO with a high deposition pressure of 1.5 mTorr and a low power supply of 100 W; 120 nm IOH). (b) EQE spectra of the champion MTJSC without MgF_2 antireflection coating. (c) Comparison of reflectance for the champion MTJSC with and without MgF_2 antireflection coating.

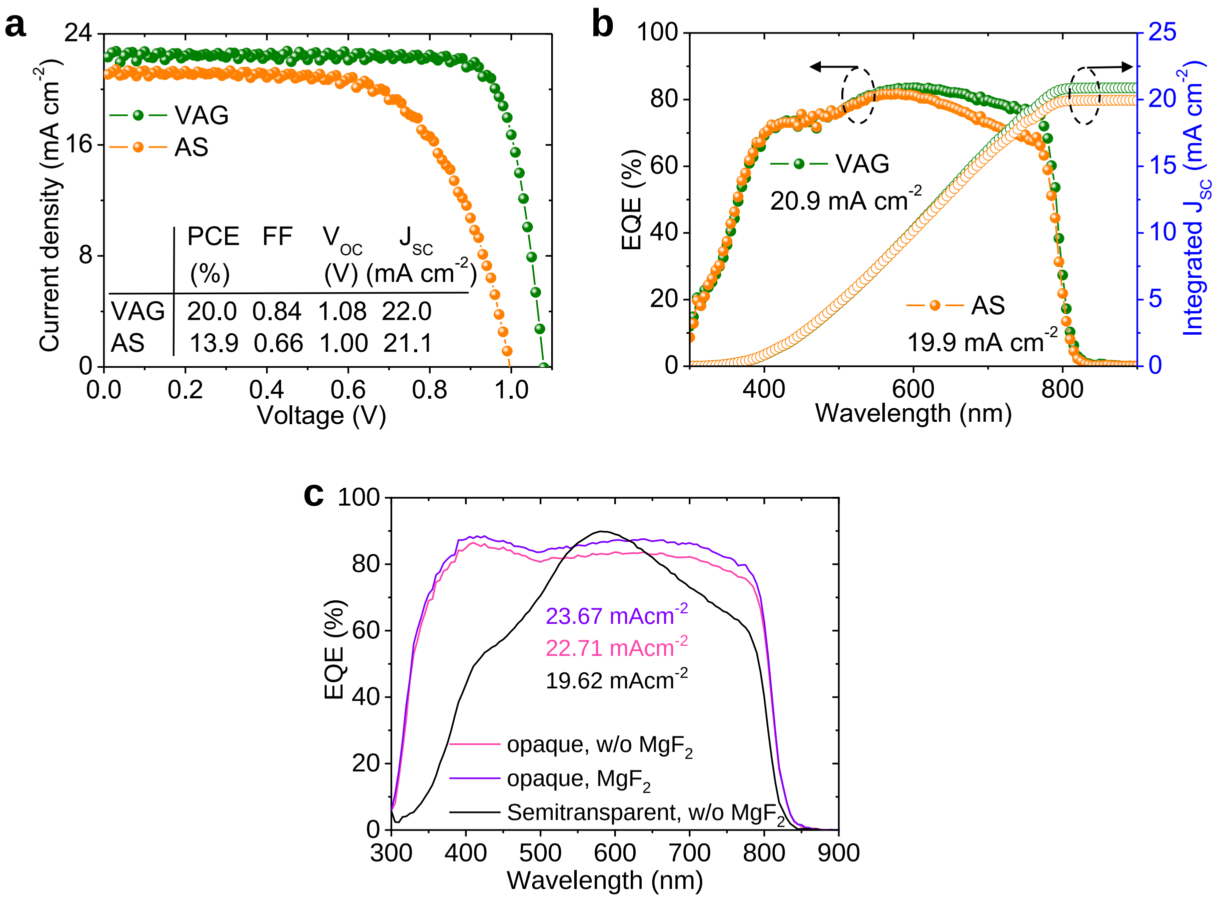

Fig. S11 PV performance of single-junction middle sub-cells. (a) *J*–*V* characteristics from backward scan and (b) EQE spectra for semitransparent devices fabricated using VAG and AS methods. (c) EQE spectra for semitransparent and opaque devices fabricated using VAG method. The semitransparent device was applied recipe 2 for TCO deposition: 165 nm IZO with a high deposition pressure of 1.5 mTorr and a low power supply of 100 W.

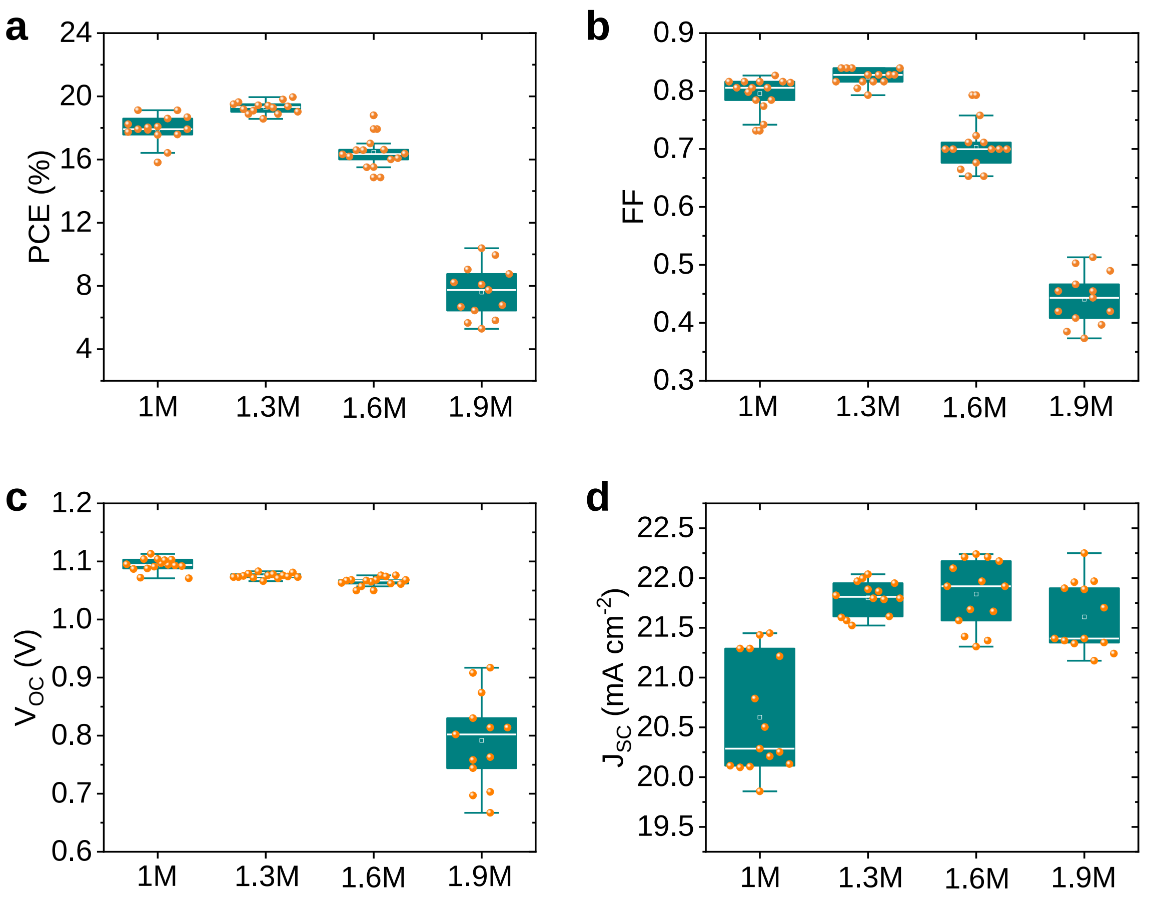

Fig. S12 Statistical distribution of the photovoltaic parameters for semitransparent single-junction middle perovskite solar cells. (a) PCE, (b) FF, (c) V_{OC}, and (d) J_{SC}. The $FAPbI_3$ thin films were fabricated by VAG method with a variation of perovskite precursor concentration from 1.0 to 1.9 M.

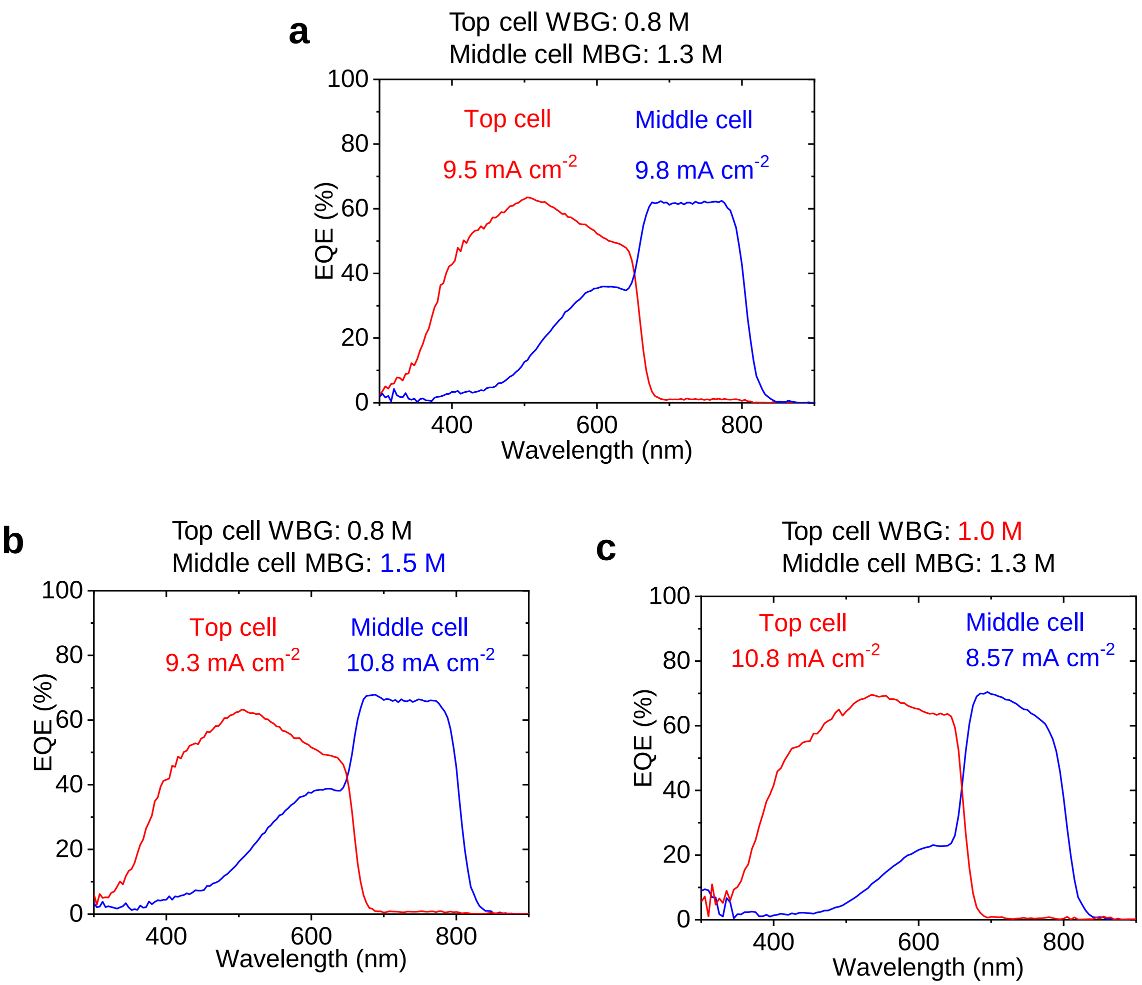

Fig. S13 EQE spectra for the MTJSCs. The WBG was fabricated by AS method with a variation of perovskite precursor concentration of 0.8 or 1.0 M. The MBG was fabricated by VAG method with a variation of perovskite precursor concentration of 1.3 or 1.5 M. TCO deposition was applied to recipe 2: 165 nm IZO with a high deposition pressure of 1.5 mTorr and a low power supply of 100 W.

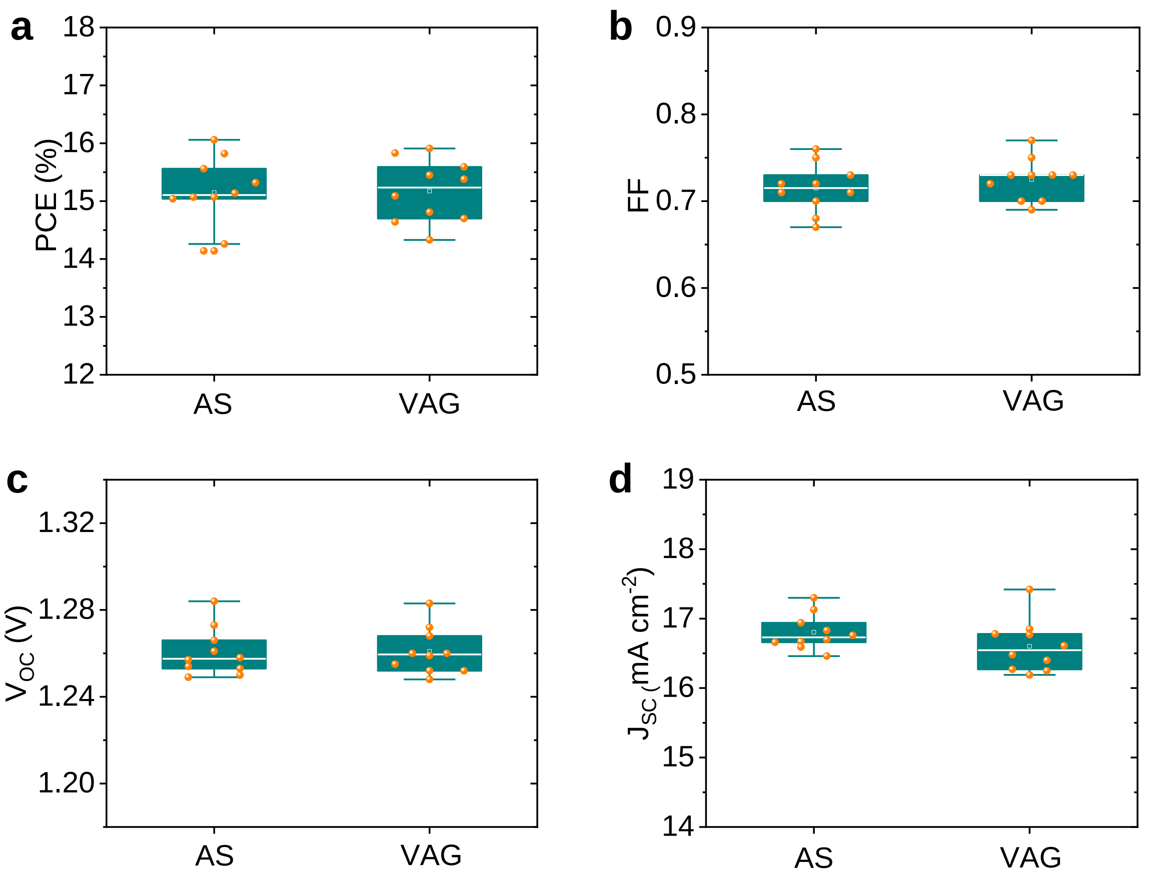

Fig. S14 Statistical distribution of the photovoltaic parameters for semitransparent single-junction top perovskite solar cells. (a) PCE, (b) FF, (c) V_{OC}, and (d) J_{SC}. The WBG thin films were fabricated by VAG or AS methods with a perovskite precursor concentration of 0.8 M.

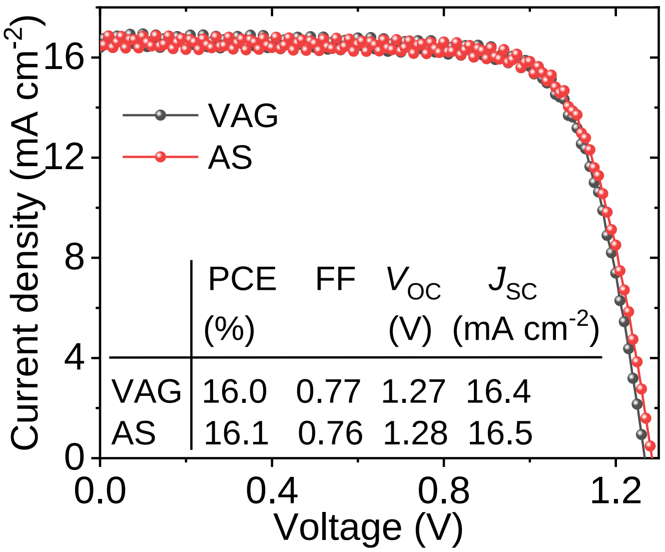

Fig. S15 *J*–*V* characteristics from the backward scan for the champion semitransparent single-junction top perovskite solar cells. The WBG perovskite thin films were fabricated by using VAG or AS methods.

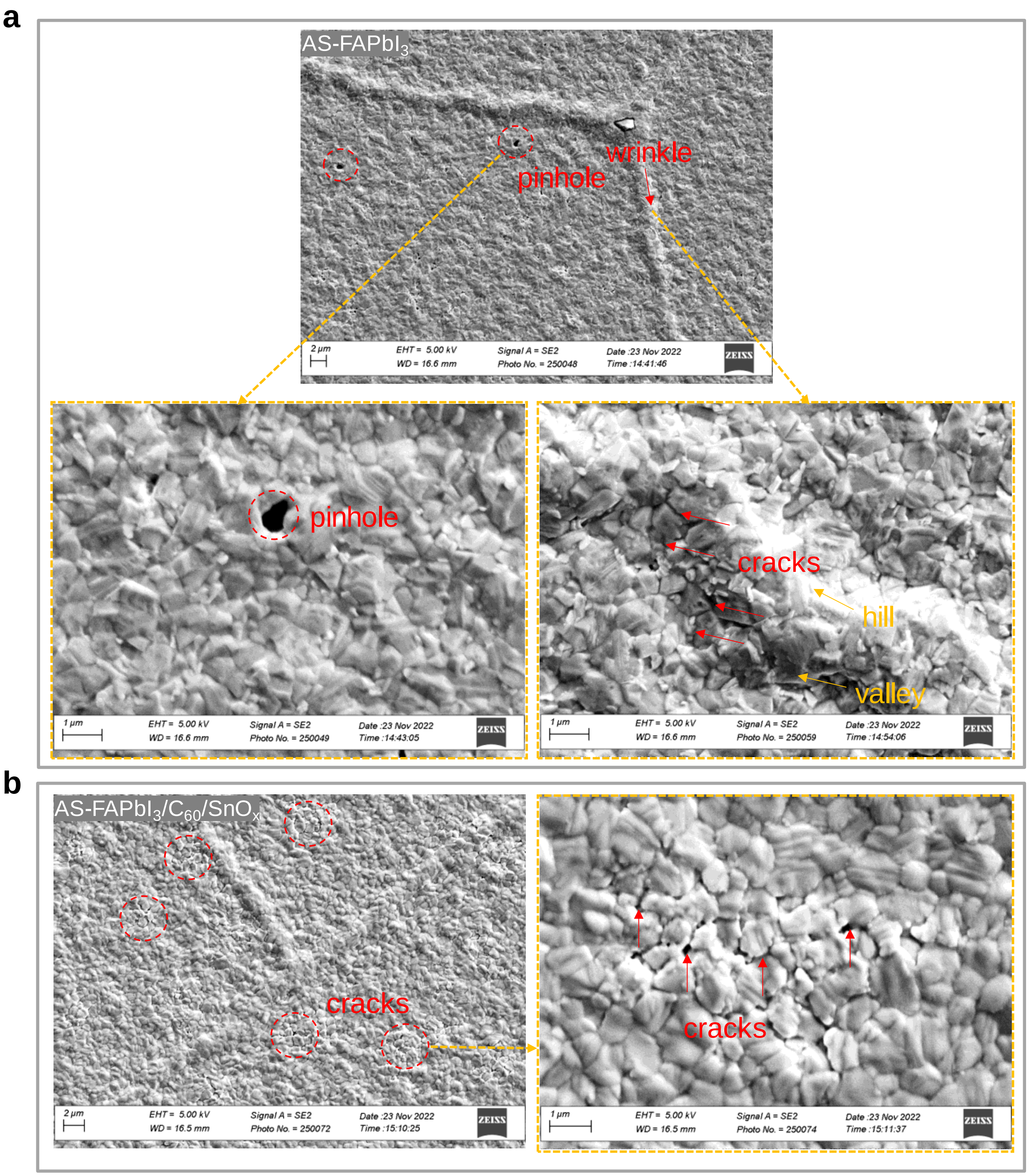

Fig. S16 Top-view SEM images of $FAPbI_3$ thin films fabricated AS methods and sequential deposition of C_{60} and SnO_x layers. (a) AS-$FAPbI_3$. (b) AS-$FAPbI_3$/C_{60}/SnO_x.

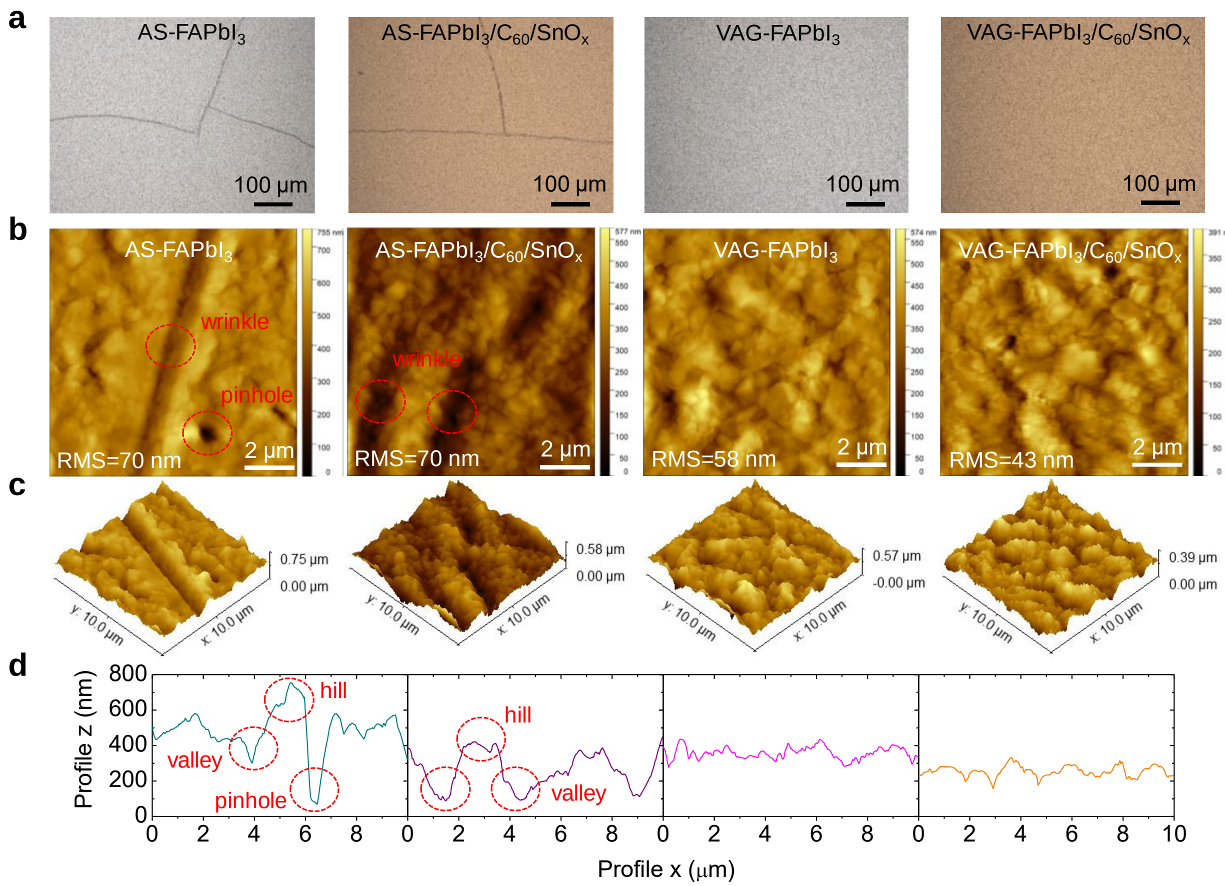

Fig. S17 The morphology of different perovskite and sequential deposition of C_{60} and SnO_x layers. (a) Optical microscopic, (b) top-view, (c) 3D AFM images, and (d) the profile of $FAPbI_3$ thin films (fabricated by VAG and AS methods) and sequential deposition of C_{60} and SnO_x layers. The pinholes observed in AS-$FAPbI_3$ thin film have a high depth of > 650 nm that is comparable with the overall perovskite thin film thickness, which causes shunting in the solar cell as well as triple-junction architecture. The pinholes and cracks allow for a faster penetration of the solvents of the WBG perovskite top cell ink.

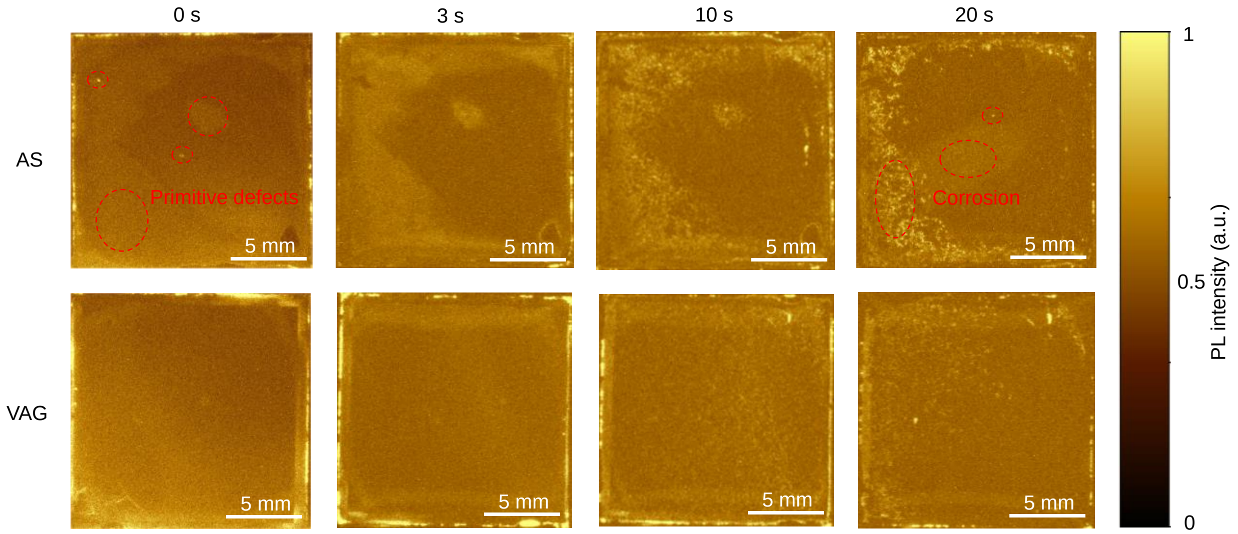

Fig. S18 PL imaging evolution *vs.* time for ITO/NiO_x/2PACz/$FAPbI_3$/C_{60}/SnO_x stack ($FAPbI_3$ thin films fabricated by VAG and AS methods). After dropping DMF on the stack, the PL images are taken at different times between 0 s and 20 s.

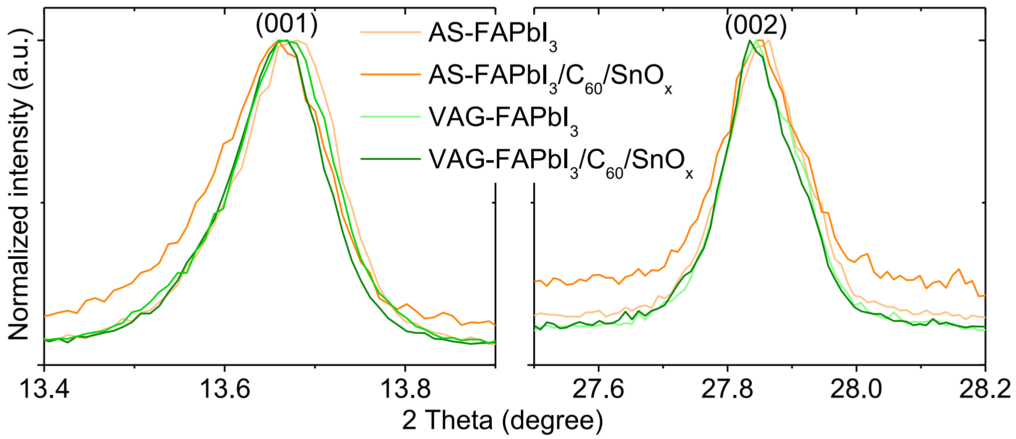

Fig. S19 Normalized intensity of XRD patterns for $FAPbI_3$ thin films (fabricated by VAG and AS methods) and sequential deposition of C_{60} and SnO_x layers.

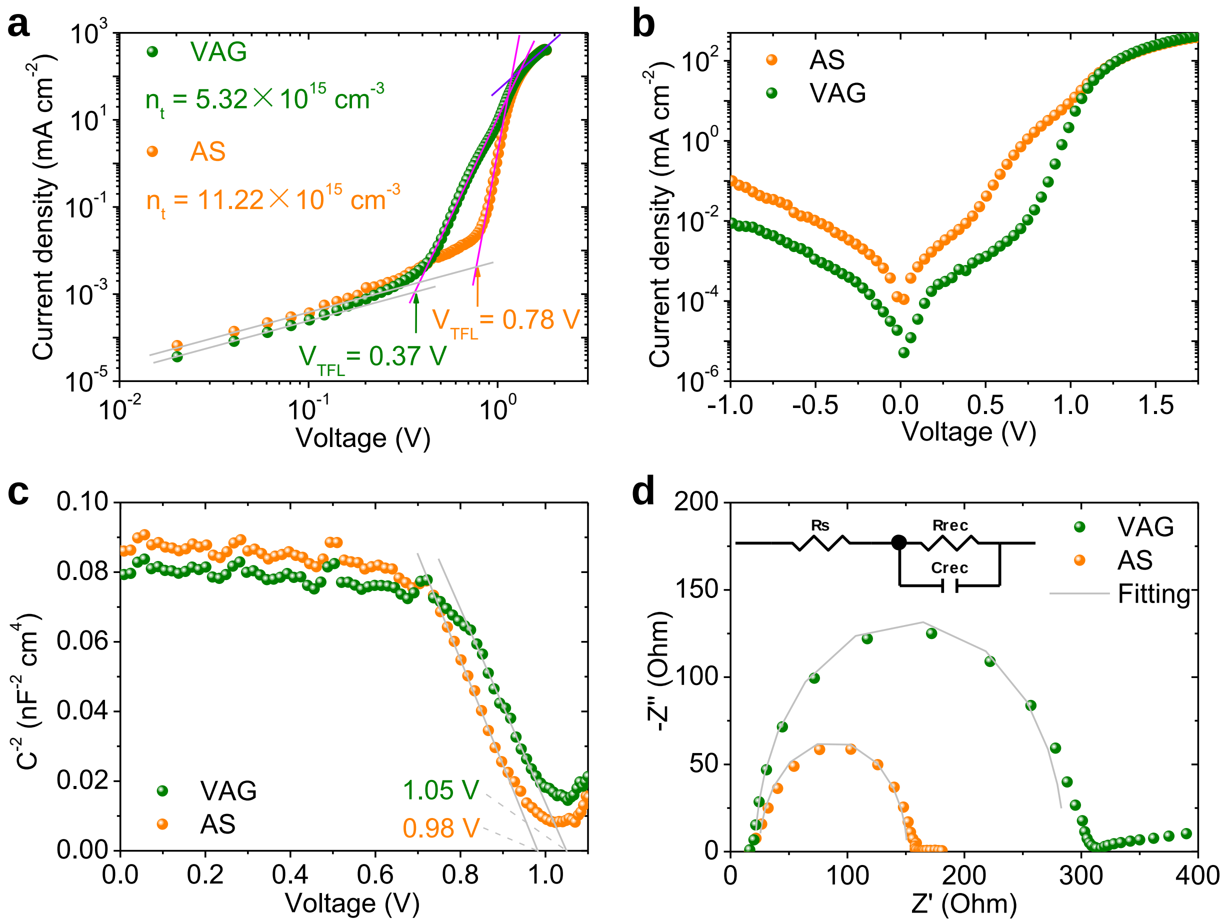

Fig. S20 Characteristics of non-radiative recombination for semitransparent middle sub-cell. (a) SCLC analyses, (b) dark *J*–*V* characteristics, (c) Mott–Schottky plots, and (d) Nyquist plots. The inset in (d) is the equivalent circuit diagram. In the low-frequency region of (d), the straight-like transmission line is related to neglected charge carrier diffusion, which is not considered for fitting.[1]

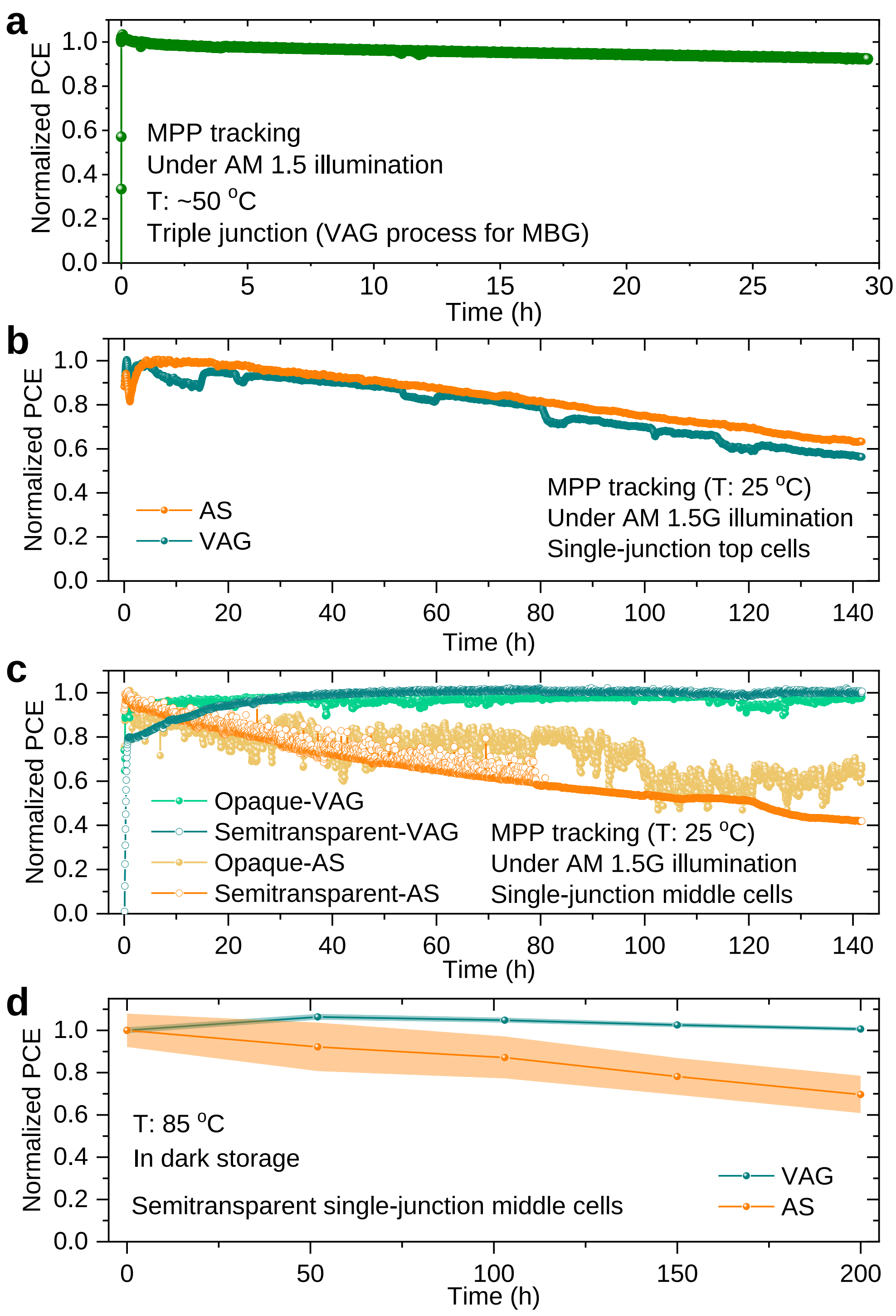

Fig. S21 Evolution of normalized PCEs. (a) perovskite–perovskite–Si MTJSC (MBG was fabricated by VAG strategy) under continuous AM 1.5G illumination at ~50 °C for MPP tracking. (b) Single-junction top cells and (c) middle cells (semitransparent and opaque architectures) under continuous AM 1.5G illumination at 25 °C for MPP tracking. (d) Semitransparent middle cells in dark storage at 85 °C. All devices without encapsulation were tested in a N_2-filled glovebox.

Table S1 Summary of different parameters for TRPL measurements.

Samples	τ_1 (ns)	Fraction 1	τ_2 (ns)	Fraction 2	t_{avg} (ns)
ITO/2PACz/$FAPbI_3$ (VAG)	-	-	-	-	3581
ITO/2PACz/$FAPbI_3$ (AS)	-	-	-	-	3313
ITO/2PACz/$FAPbI_3$/C_{60}/SnO_x (VAG)	4	0.582	27	0.418	14
ITO/2PACz/$FAPbI_3$/C_{60}/SnO_x (AS)	3	0.629	16	0.371	8

The TRPL data for ITO/2PACz/$FAPbI_3$ stack was fitted with a mono-exponential equation: $Y = A \exp(-t/\tau)$, and the data of ITO/2PACz/$FAPbI_3$/C_{60}/SnO_x stack was fitted with a biexponential equation: $Y = A_1 \exp(-t/\tau_1) + A_2 \exp(-t/\tau_2)$. τ_1 and τ_2 reveal a fast lifetime and a slow lifetime, respectively.

Table S2 Summary of different parameters for EIS measurements for $FAPbI_3$-based semitransparent single-junction solar cells.

Samples	R_s (Ω)	R_{rec} (Ω)	C_{rec} (nF)
AS	24.7	126.2	0.1
VAG	21.3	264.0	0.1

R_s: series resistance; R_{rec}: charge recombination resistance; C_{rec}: charge recombination capacitance. All data were fitted by Z-View.

References

1 H. Hu, D. B. Ritzer, A. Diercks, Y. Li, R. Singh, P. Fassl, Q. Jin, F. Schackmar, U. W. Paetzold and B. Abdollahi Nejand, *Joule*, 2023, **7**, 1–19.
